# Study on monitoring broken rails of heavy haul railway based on ultrasonic guided wave

**DOI:** 10.1038/s41598-024-59328-5

**Published:** 2024-04-15

**Authors:** Xining Xu, Ziyu Wen, Yi Ni, Bohuai Shao, Xinyu Ma, Zecheng Pan

**Affiliations:** 1 State Key Laboratory of Advanced Rail Autonomous Operation, Beijing Jiaotong University, Beijing, 100044 China; 2 School of Mechanical, Electronic, and Control Engineering, Beijing Jiaotong University, Beijing, 100044 China; 3 Railway Engineering Research Institute, China Academy of Railway Sciences Corporation Limited, Beijing, 100081 China; 4 Frontiers Science Center for Smart High-Speed Railway System, Beijing, 100044 China

**Keywords:** Heavy haul railway, Ultrasonic guided wave, Monitoring broken rails, Mode selection, Mode excitation, Mechanical engineering, Characterization and analytical techniques, Electrical and electronic engineering

## Abstract

Real-time monitoring of broken rails in heavy haul railways is crucial for ensuring the safe operation of railway lines. U78CrV steel is a common material used for heavy haul line rails in China. In this study, the semi-analytical finite element (SAFE) method is employed to calculate the dispersion curves and modal shapes of ultrasonic guided waves in U78CrV heavy steel rails. Guided wave modes that are suitable for detecting rail cracks across the entire cross-section are selected based on the total energy of each mode and the vibration energy in the rail head, web, and foot. The excitation method for the chosen mode is determined by analyzing the energy distribution of the mode shape on the rail surface. The ultrasonic guided wave (UGW) signal in the rail is analyzed using ANSYS three-dimensional finite element simulation. The group velocity of the primary mode in the guided wave signal is obtained through continuous wavelet transform to confirm the existence of the selected mode. It is validated that the selected mode can be excited by examining the similarity between the vibration shapes of a specific rail section and all modal vibration shapes obtained through SAFE. Through simulation and field verification, the guided wave mode selected and excited in this study demonstrates good sensitivity to cracks at the rail head, web, and foot, and it can propagate over distances exceeding 1 km in the rail. By detecting the reflected signal of the selected mode or the degree of attenuation of the transmitted wave, long-distance monitoring of broken rails in heavy-haul railway tracks can be achieved.

## Introduction

Heavy haul railway transportation offers substantial advantages such as large transportation capacity, high efficiency, and low transportation costs, greatly fostering the development of China's national economy^1^. China's heavy haul railway mainly uses seamless rail; the phenomenon of thermal expansion and cold contraction of seamless rail is pronounced, coupled with the wheel-rail force of heavy haul railway, it is easy to lead to rail fracture. In addition, rail top abrasion, rail core damage, rail splint clamp damage, rail welding quality, and other problems can also lead to rail fracture^2^.

Currently, rail fracture detection methods are primarily categorized into mobile detection and fixed detection. Mobile detection involves assessing rail conditions using hand-propelled rail detection trolleys or large rail inspection vehicles. The detection methods mainly consist of ultrasonic, eddy currents, and magnetic flux leakage. Within ultrasonic detection, there are piezoelectric and electromagnetic methods. China's rail detection vehicle is mainly developed based on piezoelectric ultrasonic technology. For instance, the GT-2 rail detection trolley utilizes seven ultrasonic transducers with varying angles to simultaneously emit and receive ultrasonic waves on the rail for internal crack detection^3^. Based on electromagnetic ultrasonic detection technology, Canada's Tektrend company developed the RailPro rail detection vehicle^4^, which can detect defects in the rail head and web. The rail flaw detection system developed by VIGOR company^5^ in Russia realizes the detection of rail defects. Sperry company in the United States has developed a hand-pushed rail surface crack detector^6^ and a high-speed flaw detection vehicle^7^ based on eddy current detection technology and developed a rail defect detection system combined with ultrasonic detection technology and magnetic flux leakage detection technology, with an inspection speed of 35 km/h^6^. The mobile detection method has simple operation and high detection sensitivity. The advantage of mobile detection methods lies in their simple operation and high detection sensitivity, allowing for quick identification of rail defects. However, due to technological limitations, the detection distance of mobile detection methods is limited, and some areas or corners may not be fully covered and detected. Additionally, because operations must be carried out during idle periods of train transportation, mobile detection cannot monitor the condition of rails in real-time. This, to a certain extent, affects the ability to timely detect and repair rail issues.

Fixed detection refers to the installation of specific detection equipment for continuous online monitoring of rail conditions under all weather conditions. Currently, commonly used detection methods include strain gauge, optical fiber, ultrasonic guided wave, and track circuit^8^. The detection principle of strain gauge broken rail involves judging whether the rail is broken by detecting changes in rail stress. The detection distance is generally 30–60 m^9^, which is unsuitable for long-distance broken rail detection. Moreover, this method requires calibrate the position of the zero-pressure point before sensor installation^10^, which is not applicable to established lines. The optical fiber rail breaking detection involves installing optical fibers on the rail web; when the rail breaks, the optical fiber will also break so that the optical receiver cannot receive the effective signals. This method has high detection sensitivity, but due to the fragile nature of optical fibers, its installation and maintenance process is complex and delicate. It can only be used in short-range or difficult electrical isolation situations^11,12^, which is unsuitable for long-distance rail breaking monitoring. The track circuit detects the rail breaking in real-time by monitoring the changes in rail voltage, current, and other parameters, which have high accuracy and real-time performance. However, the track circuit cannot detect the partial fracture of the rail, and its detection sensitivity is low in the section with poor track bed condition. In summary, while fixed detection methods can meet the needs of online monitoring, they still have limitations. New full-section online monitoring technologies are needed for practical applications.

Ultrasonic guided wave is a unique ultrasonic wave generated through continuous reflection, refraction, and conversion of the longitudinal and transverse wave with the medium boundary when the ultrasonic wave propagates in the waveguide media^13^. Its principle for rail breaking detection is relatively simple: when the rail is completely broken, the transducer cannot receive the guided wave signal, and when the rail is partially damaged, the energy of the guided wave signal received will attenuate^14^. Compared to other detection methods, UGW detection has advantages such as long-distance propagation, covering the entire section of the rail, fast detection speed, and its detection process does not interfere with train transportation, making it suitable for long-distance rail breaking monitoring.

Lamb^15^ discovered the existence of Lamb waves and confirmed the multimodality in the early twentieth century, many scholars have conducted in-depth research on the propagation characteristics of UGW. In 1950, Thomson first proposed to use the matrix analytical method to solve the modal response results of UGW in layered solid media^16^. In the following decades, the solution method using matrix calculation was widely used to solve the propagation characteristics of UGW^17–19^. In the twenty-first century, as the waveguide medium’s cross-section becomes more complex, the solution method through matrix calculation cannot meet the needs gradually. Scholars have proposed various ways to study the propagation characteristics of the guided wave, including the finite element method, the semi-analytical finite element method, the boundary element method, etc.^20^. Among them, using the SAFE method to solve the dispersion curve of UGW in a waveguide medium has gradually become a mature method. Many scholars have done much research based on this and have made good progress. The following articles discussed numerical methods in SHM or wave propagation analysis. In 2002, Mukdadi et al.^21^ calculated the dispersion curve of a guided wave in a multi-layer rectangular section beam structure. In 2003, Hayashi et al.^22^ solved the dispersion curve of the guided waves in a waveguide medium with an arbitrary section. In 2017, Cong et al.^23^ solved the dispersion curve of UGW in spiral fin. In 2018, Duan and Kirby^24^ in the UK combined the SAFE method with the perfect matching layer to analyze the propagation characteristics of ultrasonic guided waves in fluid-filled pipes. They developed a SAFE method in 2019 to study the propagation characteristics of ultrasonic guided waves in composite laminates^25^.

In recent years, the application of UGW in railway nondestructive testing has been fully developed. In 2009, Chong et al.^26^ used the mixed design of analytical method and finite element method to design a guided wave sensor to detect the defects inside the rail so that the guided wave can propagate a longer distance and proposed the mixed analytical method of guided wave and finite element, which is used to analyze the propagation characteristics of guided wave in the rail. In 2011, Coccia et al.^27^ solved the free vibration solution and forced vibration solution of UGW propagation at the rail head under laser excitation. In 2014, Xu et al.^28^ solved the dispersion curve of UGW in CHN60 rail using the SAFE method and analyzed four modes at low frequency. In 2015, Loveday and Long^29^ used a laser vibrometer to detect the propagation ability of each UGW mode in the rail at a low frequency on the 5 m long rail in the laboratory and selected the guided wave mode that can propagate over a long distance. In 2018, Setshedi et al.^30^ estimated rail material and geometric characteristics based on SAFE and UGW and used Poisson's ratio and three geometric parameters to represent rail contour loss. In the same year, Ramatlo et al.^31^ designed the UGW transducer for detecting rail cracks by using the SAFE method and three-dimensional finite element method combined with a mathematical optimization algorithm. In 2020, they developed a modeling framework for the ultrasonic detection of waveguides with arbitrary discontinuities to simulate the detection of multiple tracks with discontinuities by ultrasonic guided waves^32^. With the continuous development of artificial intelligence technology, many researchers have begun to apply machine learning to railways^33^. In 2022, Harsh Mahajan et al.^34^ explored the application of UGW generated by pasting a piezoelectric sensor on the rail surface in high-frequency rail damage detection and proposed a machine learning algorithm framework for rail head damage detection. The experimental results show that the maximum error range of the algorithm framework in predicting damage is 2% to 16.16%. In 2022, Sresakoolchai et al.^35^ utilized track geometry data obtained by a track geometry car (TGC) to detect rail, switch and crossing, fastener, and rail joint defects. They developed accurate detection models by employing supervised and unsupervised machine learning techniques.

U78CrV rail is a wear-resistant heavy haul railway developed by Angang Steel Group Co. LTD. With good comprehensive performance, it has been widely laid and used in China since 2009^36^. It is one of China's most commonly used rail materials for heavy haul railways. Currently, there is a lack of mature and reliable online broken rail monitoring system for the long-distance broken rail monitoring problem in heavy-haul railways. However, the advantages of ultrasonic guided wave testing, such as long detection distance and fast detection speed, make it have broad application prospects in the field of long-distance broken rail detection.

In recent years, the laboratory team has conducted extensive research in detecting rail fractures and rail stress using UGW^14,20,28,35–38^. Aiming at the application of UGW in the rail break detection of heavy haul railways in China, this paper combines the semi-analytical finite element method and three-dimensional finite element method to identify a guided wave mode suitable for the long-distance detection of broken rails made of U78CrV type heavy rail, as well as the excitation method for the selected mode. It is demonstrated that the excitation method can successfully induce the chosen mode by verifying the consistency of group velocity and the similarity of vibration shape. Simulation experiments show that the selected mode exhibits good sensitivity to the rail head, rail web, and rail foot. Finally, an attenuation test confirms that the chosen mode is suitable for long-distance rail break detection in heavy haul railways.

## Dispersion curve of guided wave in U78CrV rail

**Figure 1:**
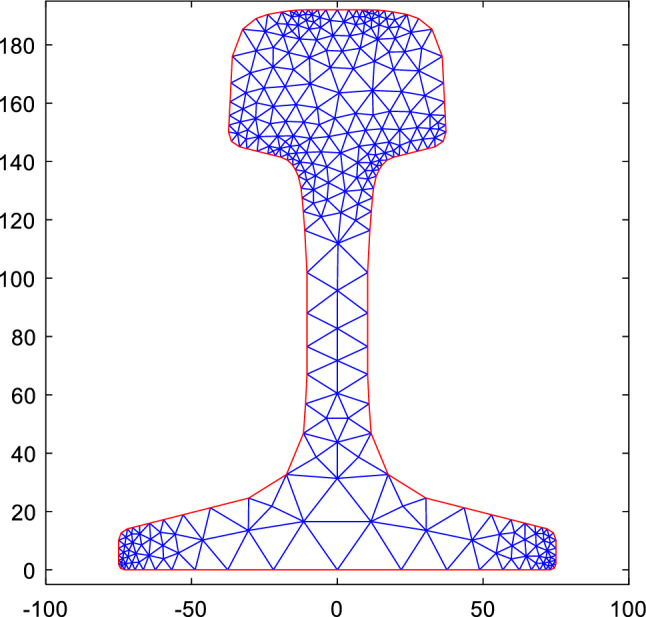
Discrete drawing of 75kg/m rail section.

According to the dimensional specifications of the 75 kg/m rail, the cross-section of the rail is drawn in SolidWorks. The rail section height is 192 mm, the rail head width is 75 mm, and the rail foot width is 150 mm. The coordinate data of the section is imported into MATLAB and plotted to represent the rail cross-section Ω. The cross-section is then discretized into triangular grid elements **Ω**_**e**_ using the PDE discretization tool of MATLAB. The division effect is shown in Fig. 1. The section is divided into 550 units consisting of 340 nodes.

The direction of guided wave propagation is defined as the z-direction, and the rail section is defined as the Oxy plane, where the y-direction represents the vertical direction. According to the SAFE theory^38^, the harmonic displacement, stress, and strain field components of each point in the rail can be expressed as:

1 $$\begin{aligned} {\mathbf{u}} & = \left[ {u_{x} u_{y} u_{z} } \right]^{T} \\ {{\varvec{\upsigma}}} & = \left[ {\sigma_{x} \sigma_{y} \sigma_{z} \sigma_{yz} \sigma_{xz} \sigma_{xy} } \right]^{T} \\ {{\varvec{\upvarepsilon}}} & = \left[ {\varepsilon_{x} \varepsilon_{y} \varepsilon_{z} \gamma_{yz} \gamma_{xz} \gamma_{xy} } \right]^{T} \\ \end{aligned}$$

The relationship between stress and strain is given by **σ = C ε**, and **C** is the constant elastic matrix of rail.

The displacement field can express the strain at any point in the rail as:

2 $${{\varvec{\upvarepsilon}}} = \left[ {{\mathbf{L}}_{x} \frac{\partial u}{{\partial x}} + {\mathbf{L}}_{y} \frac{\partial u}{{\partial y}} + {\mathbf{L}}_{z} \frac{\partial u}{{\partial z}}} \right]$$

where:

$${\mathbf{L}}_{x} = \left[ {\begin{array}{*{20}c} 1 & 0 & 0 \\ 0 & 0 & 0 \\ 0 & 0 & 0 \\ 0 & 1 & 0 \\ 0 & 0 & 0 \\ 0 & 0 & 1 \\ \end{array} } \right],\quad {\mathbf{L}}_{y} = \left[ {\begin{array}{*{20}c} 0 & 0 & 0 \\ 0 & 1 & 0 \\ 0 & 0 & 0 \\ 1 & 0 & 0 \\ 0 & 0 & 1 \\ 0 & 0 & 0 \\ \end{array} } \right],\quad {\mathbf{L}}_{z} = \left[ {\begin{array}{*{20}c} 0 & 0 & 0 \\ 0 & 0 & 0 \\ 0 & 0 & 1 \\ 0 & 0 & 0 \\ 0 & 1 & 0 \\ 1 & 0 & 0 \\ \end{array} } \right]$$

The amplitude variation of the section displacement field can be expressed by Eq. (3).

3 $${\mathbf{u}}\left( {x,y,z,t} \right) = \left[ {\begin{array}{*{20}c} {u_{x} \left( {x,y,z,t} \right)} \\ {u_{y} \left( {x,y,z,t} \right)} \\ {u_{z} \left( {x,y,z,t} \right)} \\ \end{array} } \right] = \left[ {\begin{array}{*{20}c} {U_{x} \left( {x,y} \right)} \\ {U_{y} \left( {x,y} \right)} \\ {U_{z} \left( {x,y} \right)} \\ \end{array} } \right]{\text{e}}^{{{\text{i}}\left( {\xi z - \omega t} \right)}}$$

where *ξ* is the wave number in the *z*-direction, and *ω* is the angular frequency.

According to Eq. (4), the displacement of any point within the discrete triangular element can be expressed using the shape function as:

4 $${\mathbf{u}}^{\left( e \right)} \left( {x,y,z,t} \right)\left\lfloor { = \begin{array}{*{20}c} {\mathop \sum \limits_{k = 1}^{3} N_{k} \left( {x,y} \right)U_{xk} } \\ {\mathop \sum \limits_{k = 1}^{3} N_{k} \left( {x,y} \right)U_{yk} } \\ {\mathop \sum \limits_{k = 1}^{3} N_{k} \left( {x,y} \right)U_{zk} } \\ \end{array} } \right\rfloor^{\left( e \right)} e^{{{\text{i}}\left( {\xi z - \omega t} \right)}} = {\mathbf{N}}\left( {x,y} \right){\mathbf{q}}^{\left( e \right)} e^{{i\left( {\xi z - \omega t} \right)}}$$

where **N**(*x*, *y*) is the shape function matrix, **q**^(*e*)^ is the node displacement vector. The shape function of each node can be calculated from the coordinates of the node in the section^39^. Based on the Hamiltonian principle, the general homogeneous wave equation of UGW can be deduced^39^:

5 $$\begin{array}{*{20}c} {\left[ {{\varvec{K}}_{1} + i\xi {\varvec{K}}_{2} + \xi^{2} {\varvec{K}}_{{}} - \omega^{2} {\varvec{M}}} \right]U = 0} \\ \end{array}$$

In the equations, **K**_1_, **K**_2_, **K**_3_ are three stiffness matrices of size 3n × 3n, where n is the total number of nodes. These matrices can be calculated directly according to the material parameters of U78CrV. **M** and **U** are the mass matrix and displacement matrix, respectively. The wave equation precisely describes the dispersion characteristics and multimodality of UGW. By introducing an auxiliary matrix of size 3n × 3n, the imaginary part in Eq. (5) can be eliminated.

$${\mathbf{T}} = \left[ {\begin{array}{*{20}c} i & {} & {} & {} & {} & {} & {} \\ {} & 1 & {} & {} & {} & {} & {} \\ {} & {} & 1 & {} & {} & {} & {} \\ {} & {} & {} & \ddots & {} & {} & {} \\ {} & {} & {} & {} & i & {} & {} \\ {} & {} & {} & {} & {} & 1 & {} \\ {} & {} & {} & {} & {} & {} & 1 \\ \end{array} } \right]$$

After introducing the auxiliary matrix, Eq. (5) becomes:

6 $$\begin{array}{*{20}c} {\left[ {{\varvec{K}}_{1} + \xi \hat{\user2{K}}_{2} + \xi^{2} {\varvec{K}}_{3} - \omega^{2} {\varvec{M}}} \right]\hat{\user2{U}} = 0} \\ \end{array}$$

In the equations, $\hat{\user2{K}}_{2} = \frac{{{\varvec{T}}^{{\text{T}}} {\varvec{K}}_{2} {\varvec{T}}}}{ - i},\hat{\user2{U}} = {\varvec{TU}}$. Equation (7) can be reformulated as a first-order eigenvalue system:

7 $$\begin{array}{*{20}c} {\left[ {{\varvec{A}} - \xi {\varvec{B}}} \right]\left[ {\begin{array}{*{20}c} {\hat{\user2{U}}} \\ {\xi \hat{\user2{U}}} \\ \end{array} } \right] = 0} \\ \end{array}$$

In the equations:

$$\begin{aligned} {\varvec{A}} & = \left[ {\begin{array}{*{20}c} 0 & {{\varvec{K}}_{1} - \omega^{2} {\varvec{M}}} \\ {{\varvec{K}}_{1} - \omega^{2} {\varvec{M}}} & {\hat{\user2{K}}_{2} } \\ \end{array} } \right], \\ {\varvec{B}} & = \left[ {\begin{array}{*{20}c} {{\varvec{K}}_{1} - \omega^{2} {\varvec{M}}} & 0 \\ 0 & { - {\varvec{K}}_{3} } \\ \end{array} } \right] \\ \end{aligned}$$

For each *ω*, we can obtain 6n eigenvalues of the wavenumber *ξ* from Eq. (7). Among these, the real eigenvalues correspond to propagating guided wave modes, while the complex eigenvalues correspond to evanescent waves. By iterating through the values of *ω*, we can obtain the *ξ* − *ω* relationship, from which we can solve for the phase velocity dispersion curve of ultrasonic guided waves. The phase velocity *Cp* is defined as follows:

8 $$\begin{array}{*{20}c} {C_{p} = \frac{\omega }{\xi }} \\ \end{array}$$

**Figure 2:**
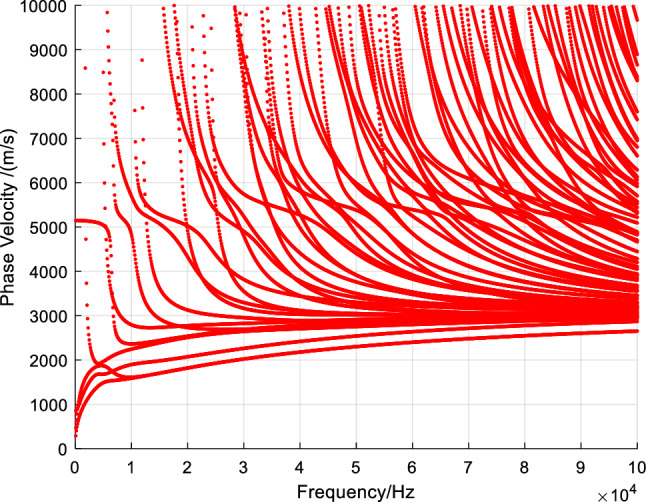
Dispersion curve of phase velocity in U78CrV rail.

Based on Eq. (8), we can obtain the phase velocity dispersion curve of ultrasonic guided waves in the cross-section of U78CrV steel rails, as shown in Fig. 2.

From the dispersion curve, it can be observed that there are multiple guided wave modes in the steel rail. As the frequency of the guided waves changes, the modes of the guided waves also change. Moreover, with an increase in frequency, the number of guided wave modes increases.

Group velocity is defined as the derivative of angular frequency to wave number :

9 $$\begin{array}{*{20}c} {C_{g} = \frac{d\omega }{{d\xi }}} \\ \end{array}$$

**Figure 3:**
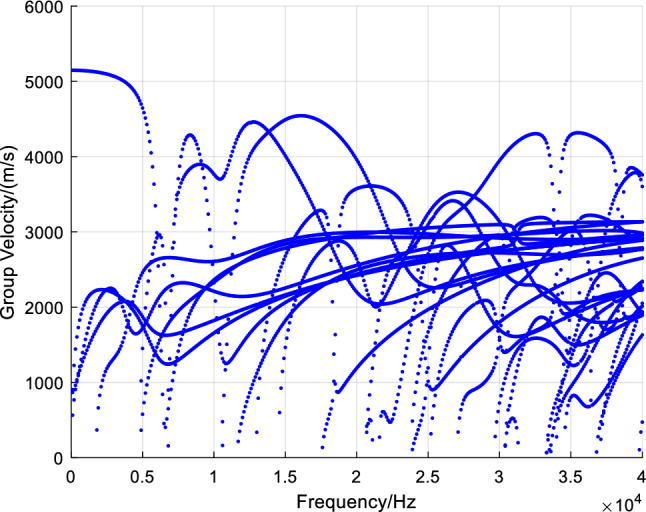
Group velocity dispersion curve in U78CrV rail.

And finally, the calculation formula of group velocity is^28^:

10 $$\begin{array}{*{20}c} {C_{g} = \frac{\partial \omega }{{\partial \xi }} = \frac{{{\hat{\mathbf{U}}}_{L}^{T} \left( {{\hat{\mathbf{K}}}_{2} + 2\xi {\mathbf{K}}_{3} } \right){\hat{\mathbf{U}}}_{R} }}{{2\omega {\hat{\mathbf{U}}}_{L}^{T} {\mathbf{M}}{\hat{\mathbf{U}}}_{R} }}} \\ \end{array}$$

where ${\hat{\mathbf{K}}}_{2}$ is a symmetric matrix for undamped motion, ${\hat{\mathbf{U}}}$ is a new nodal displacement vector, ${\hat{\mathbf{U}}}_{R}$ represents the right eigenvector and $\hat{\user2{U}}_{L}$ represents the left eigenvector $\omega , \xi , {\hat{\mathbf{U}}}_{L} ,{\hat{\mathbf{U}}}_{R}$ can be obtained when solving the characteristic equation. The dispersion curve of guided wave group velocity in U78CrV rail is obtained by taking them into Eq. (10), as shown in Fig. 3.

**Figure 4:**
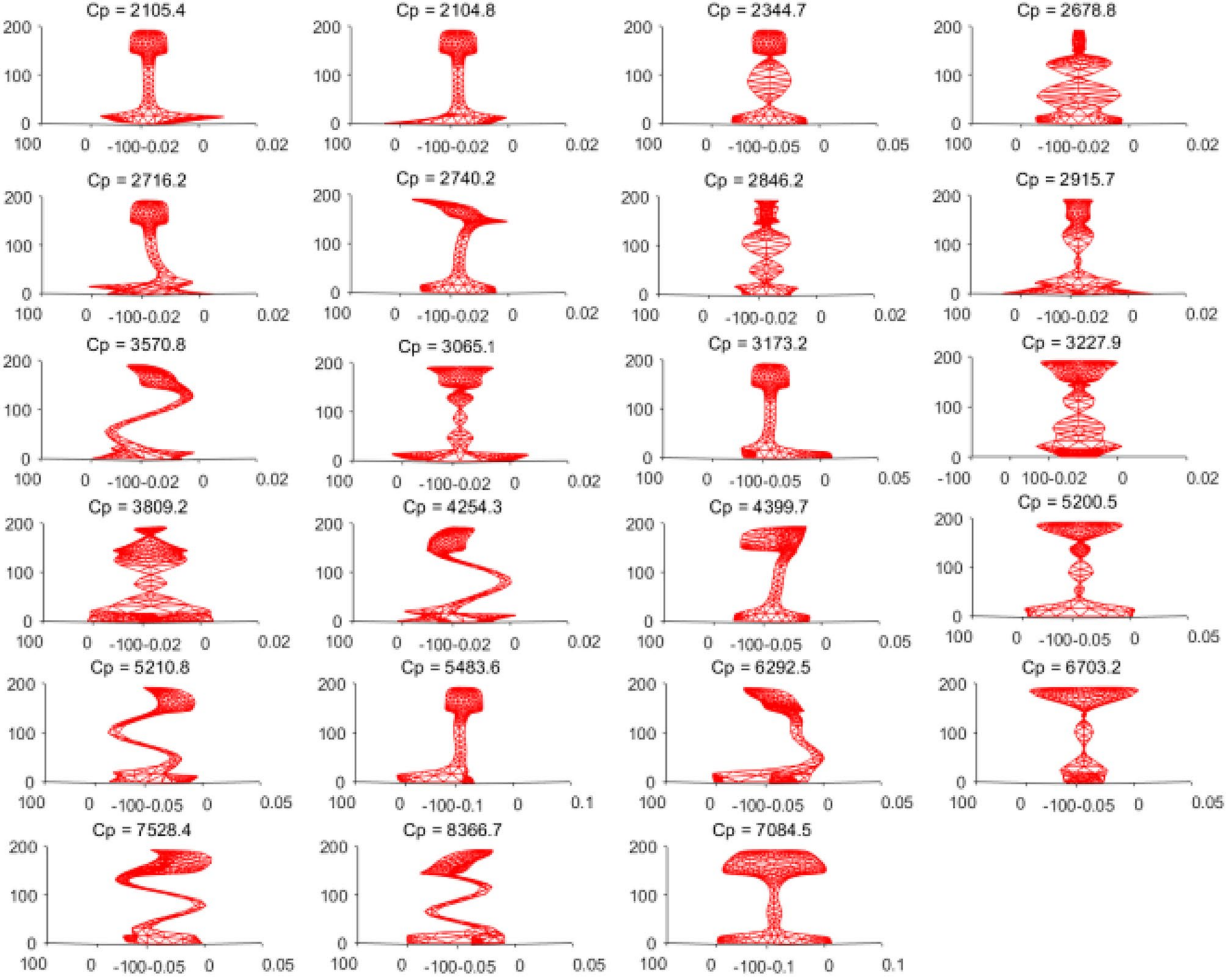
Guided wave modes in rails at 35 kHz.

Based on the node displacement information in the eigenvectors solved in the previous step, the vibration mode shapes of all guided wave modes can be plotted. Taking the guided wave frequency of 35kHz as an example, there are 23 propagation modes in the rail at this frequency, and the vibration shape diagram of each mode is shown in Fig. 4.

## Mode selection

According to the previous research^40^, when using UGW to detect the long-distance rail breakage of U78CrV rail, selecting 35kHz as the guided wave frequency has a good detection effect. To ensure that the exciting mode can cover the whole section of the rail, the mode requires a large vibration amplitude at the rail’s head, web, and foot.

At the frequency of 35kHz, there are 23 guided wave modes in the rail. The rail section height of 138–192 mm is defined as the rail head area, 33–138 mm as the rail web area, and 0–33 mm as the rail foot area. Define the vibration displacement of the No. **i** node of mode *m* as ($u_{xi}^{m} ,u_{yi}^{m} ,u_{zi}^{m}$), and the average vibration energy of mode *m* is defined as shown in Eq. (8).

11 $$\begin{array}{*{20}c} {E^{m} = \frac{1}{n}\mathop \sum \limits_{i = 1}^{n} \left( {u_{xi}^{m2} + u_{yi}^{m2} + u_{zi}^{m2} } \right)} \\ \end{array}$$

**Figure 5:**
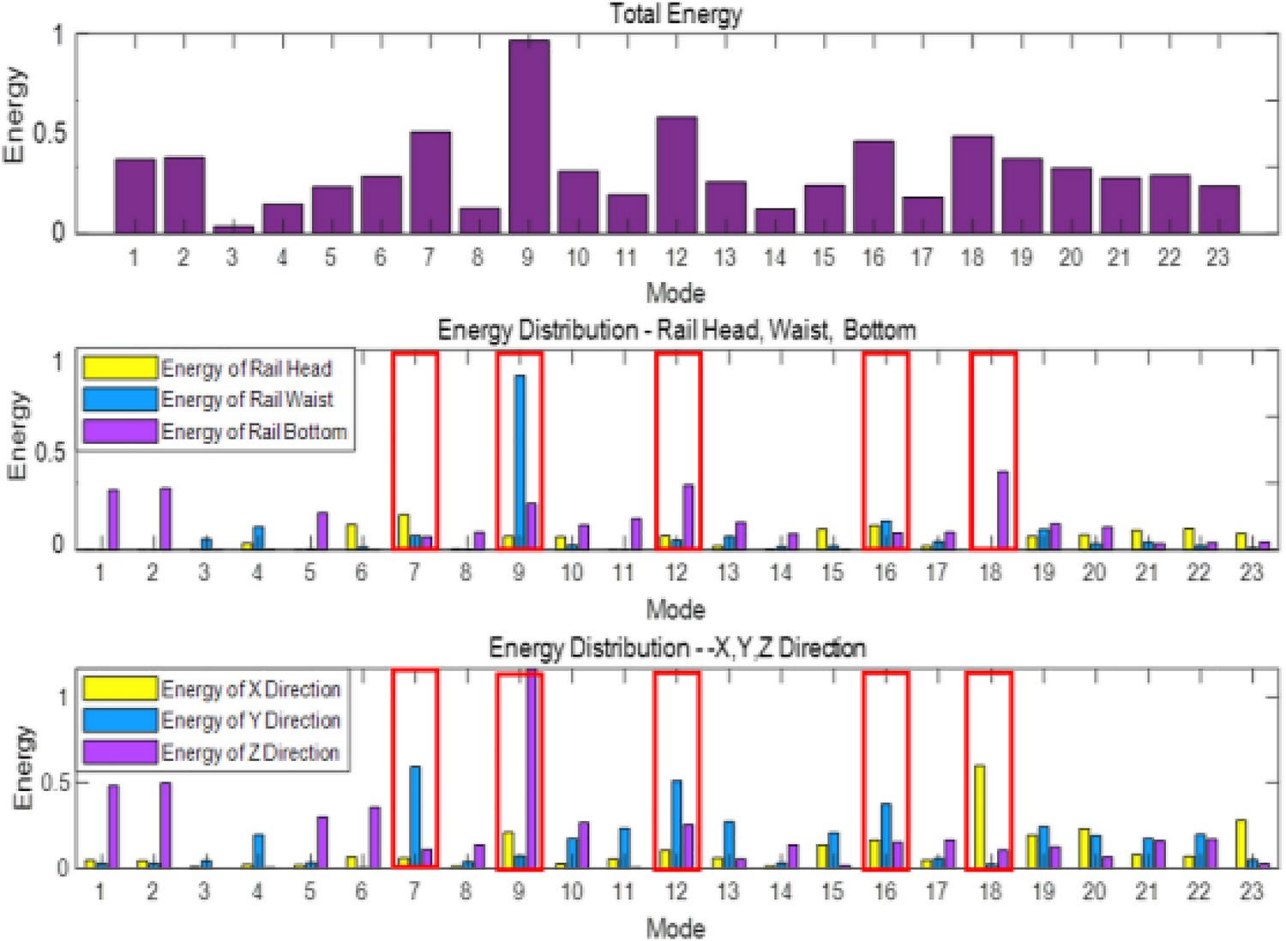
Vibration energy distribution of 23 guided wave modes.

According to the vibration data of all nodes in each area, the average vibration energy of 23 modes at the rail head, rail web, and rail foot, as well as the vibration energy of each mode in *x*, *y*, and *z*-directions are calculated respectively. The corresponding results are presented in the form of histograms, as shown in Fig. 5.

**Figure 6:**
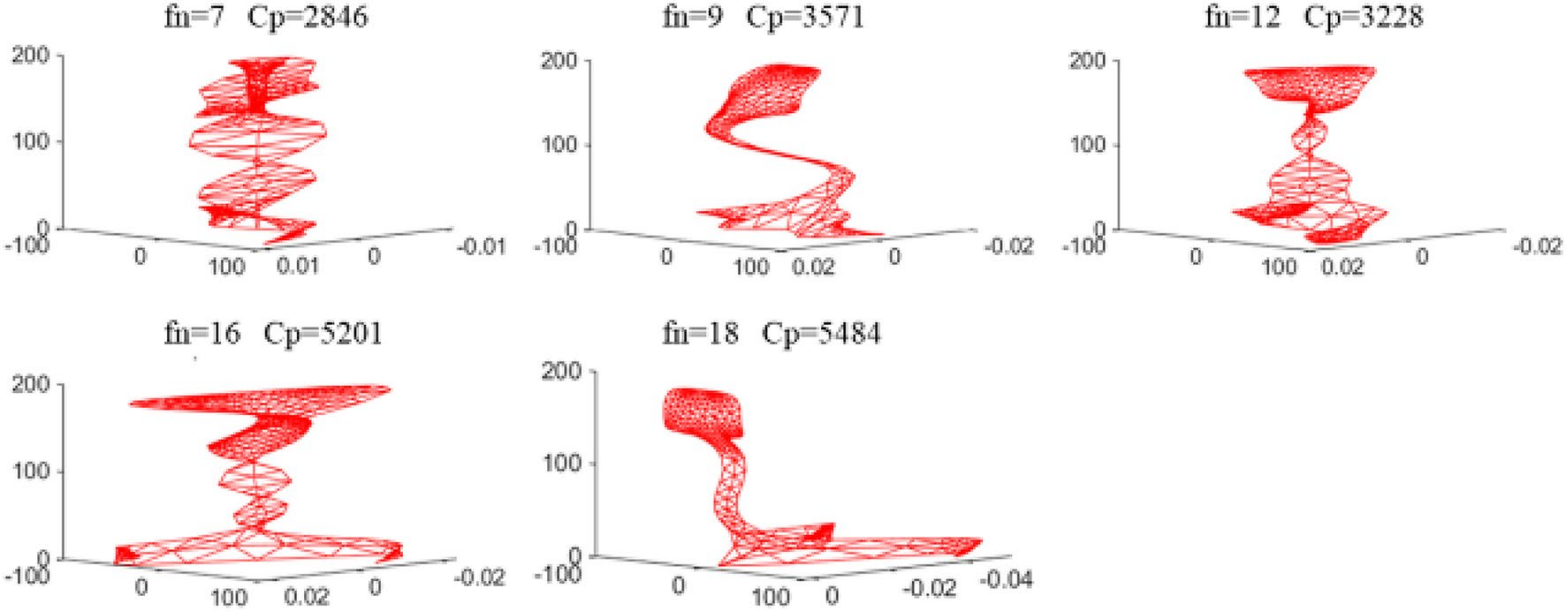
Vibration shapes diagram of the selected five modes.

We selected the five modes with the highest total energy for in-depth analysis. Theses modes are mode 7, mode 9, mode 12, mode 16 and mode 18. The mode shapes are shown in Fig. 6, where Cp is the phase velocity of the mode in m/s.

**Figure 7:**
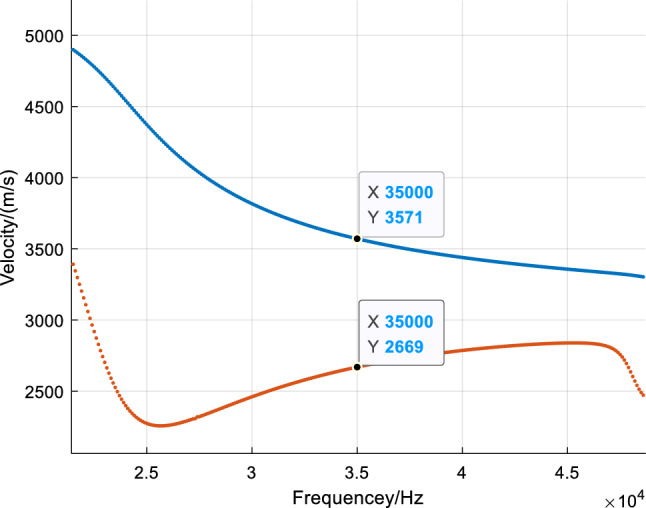
Phase velocity and group velocity dispersion curve of mode 9.

Observing the vibration shapes of the five modes, it can be seen that mode 7, mode 12, and mode 16 exhibit many distorted forms, making them difficult to excite in the field environment. Additionally, the energy of mode 18 is very limited at the rail head and rail web, which may result in the omission of defects. Therefore, mode 9 is finally selected. The modes in the dispersion curve are separated, and the phase velocity and group velocity dispersion curves of mode 9 are drawn separately, as shown in Fig. 7. The blue line and red line in the figure represent the phase velocity and group velocity curves of mode 9, respectively. When the frequency is 35kHz, the phase velocity and group velocity of mode 9 solved by SAFE are 3571m/s and 2669m/s, respectively.

**Figure 8:**
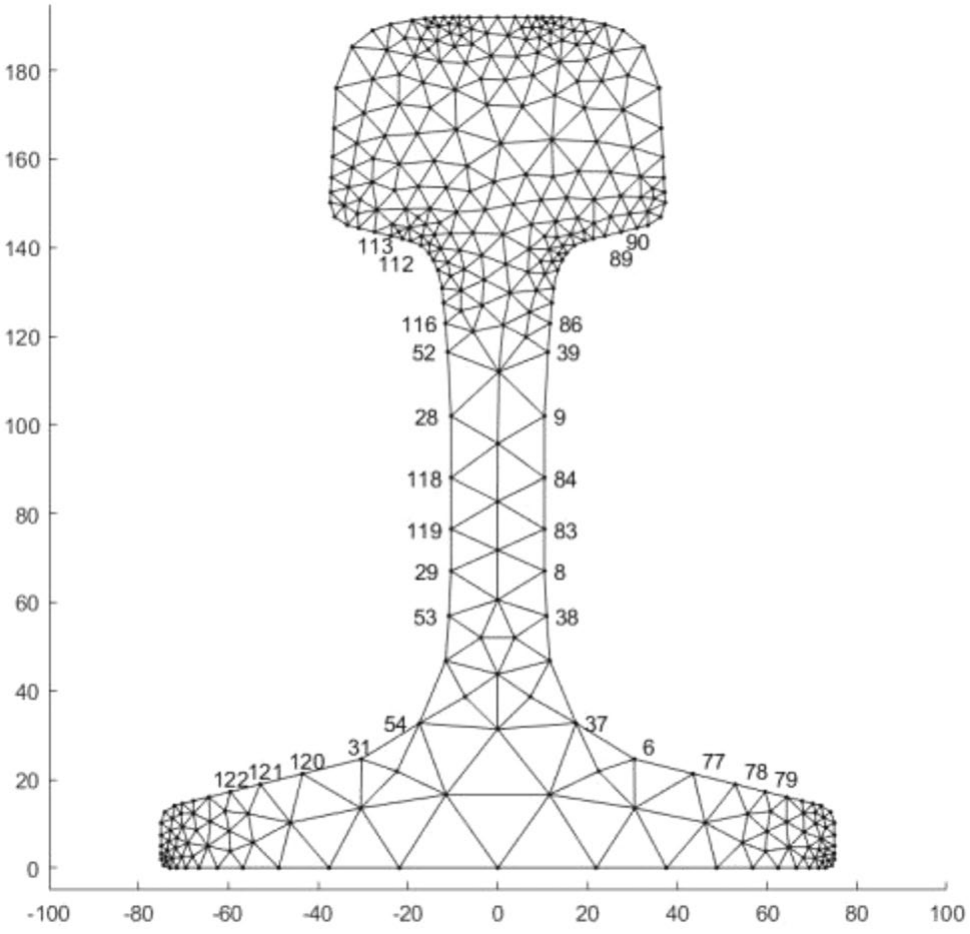
The node position where the transducer can be installed.

**Figure 9:**
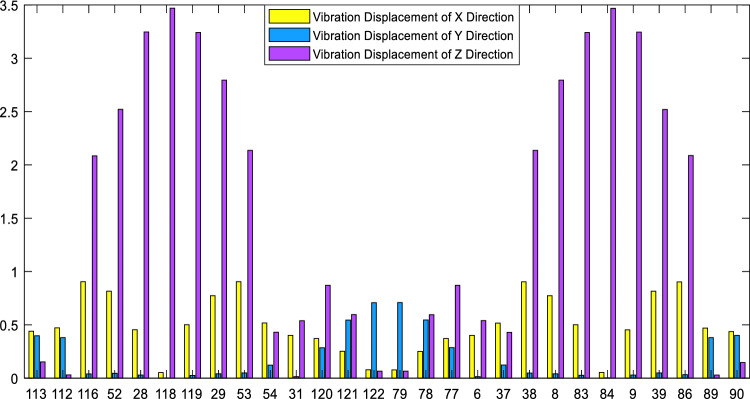
Vibration displacement of mode 9 at each node.

According to the field environment, the nodes that the UGW transducer can be installed on the outer surface of the rail section are selected, as shown in Fig. 8. There are 28 nodes symmetrically on the rail surface that meet the requirements. To choose the best excitation point, the vibration displacement of mode 9 in the *x*, *y*, and *z*-directions at 28 nodes is calculated, and the histogram is plotted, as shown in Fig. 9. The point with a large displacement is selected as the excitation point. It can be seen from the figure that the displacement of mode 9 in the z direction is the largest at nodes 118 and 84. Select node 84 (*x* = 10.4mm, *y* = 88mm) to excite in the *z*-direction (longitudinal direction of the rail). The following ANSYS simulation analysis is conducted to verify whether mode 9 can be excited here.

## Mode verification

ANSYS is a commonly used three-dimensional simulation and analysis software. The rail vibration data are obtained through the transient dynamic simulation calculation of ANSYS, the group velocity is solved by wavelet transform, and the vibration shape of the rail section is analyzed to determine the guided wave mode in the vibration signal.

### Mode verification based on group velocity

**Figure 10:**
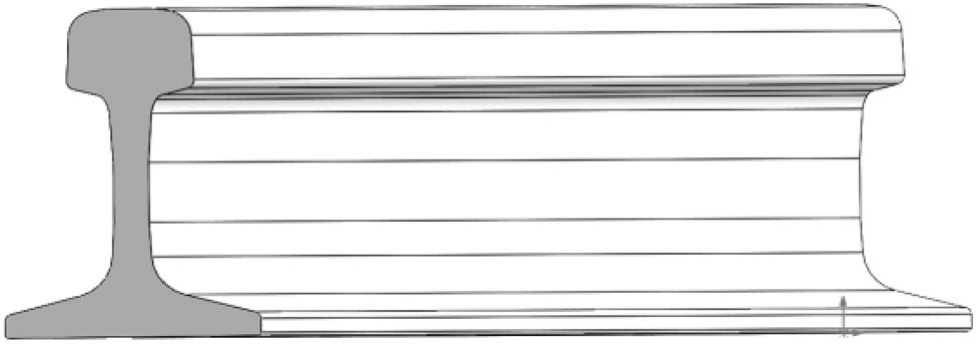
3D model of 5m long U78CrV rail.

A 5m long three-dimensional rail model is established by SolidWorks, as shown in Fig. 10.

**Figure 11:**
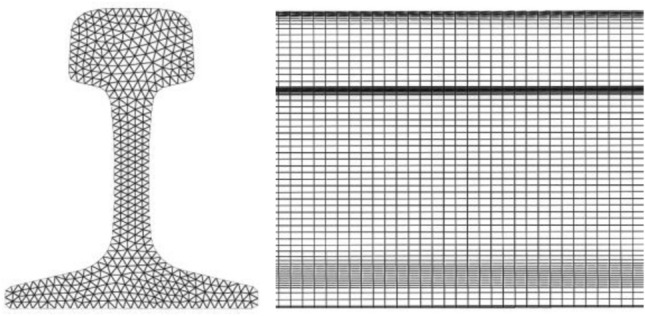
3D mesh generation effect of rail.

The rail model is meshed using Hypermesh software, and each section is divided into 480 nodes. The meshing effect is shown in Fig. 11.

The material properties of the U78CrV rail are assigned to all grid units, and the rail material parameters are shown in Table 1.

**Table 1:** Material parameters of U78CrV rail.

Model	Density (kg/m^3^)	Young's modulus (Pa)	Poisson's ratio
U78CrV	7850	2.08e11	0.33

**Figure 12:**
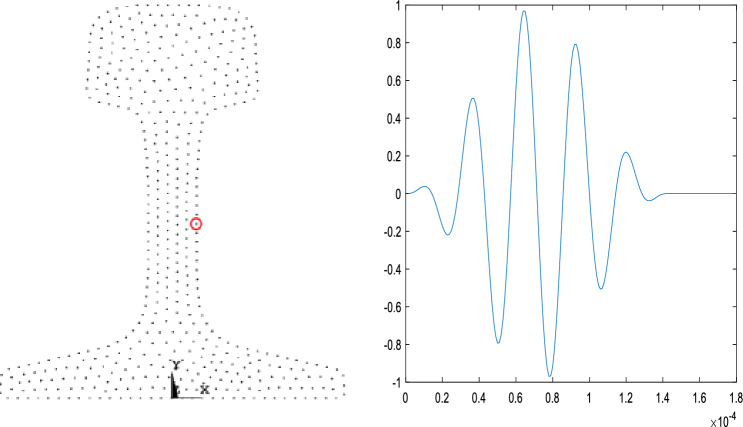
Excitation position (the left) and excitation signal (the right).

The established mesh model is imported into the ANSYS software for transient dynamic analysis. During the simulation, the element type used is the 3D solid element SOLID45 with 8 nodes. The material properties are as follows: material density is 7850 kg/m^3^, elastic modulus E is 208 GPa, Poisson's ratio ν is 0.33. The material is modeled as an isotropic material, MAT1, with properties that do not change with temperature. The excited ultrasonic guided wave modes are analyzed using ANSYS. According to the analysis results in section "Mode selection", at the middle of the rail web(*x* = 10.4mm, *y* = 88mm), a five-cycle sinusoidal signal modulated by the Hanning window is applied longitudinally along the rail, as shown in Fig. 12. The left figure shows the position of the excitation point and the right figure shows the excitation signal.

**Figure 13:**
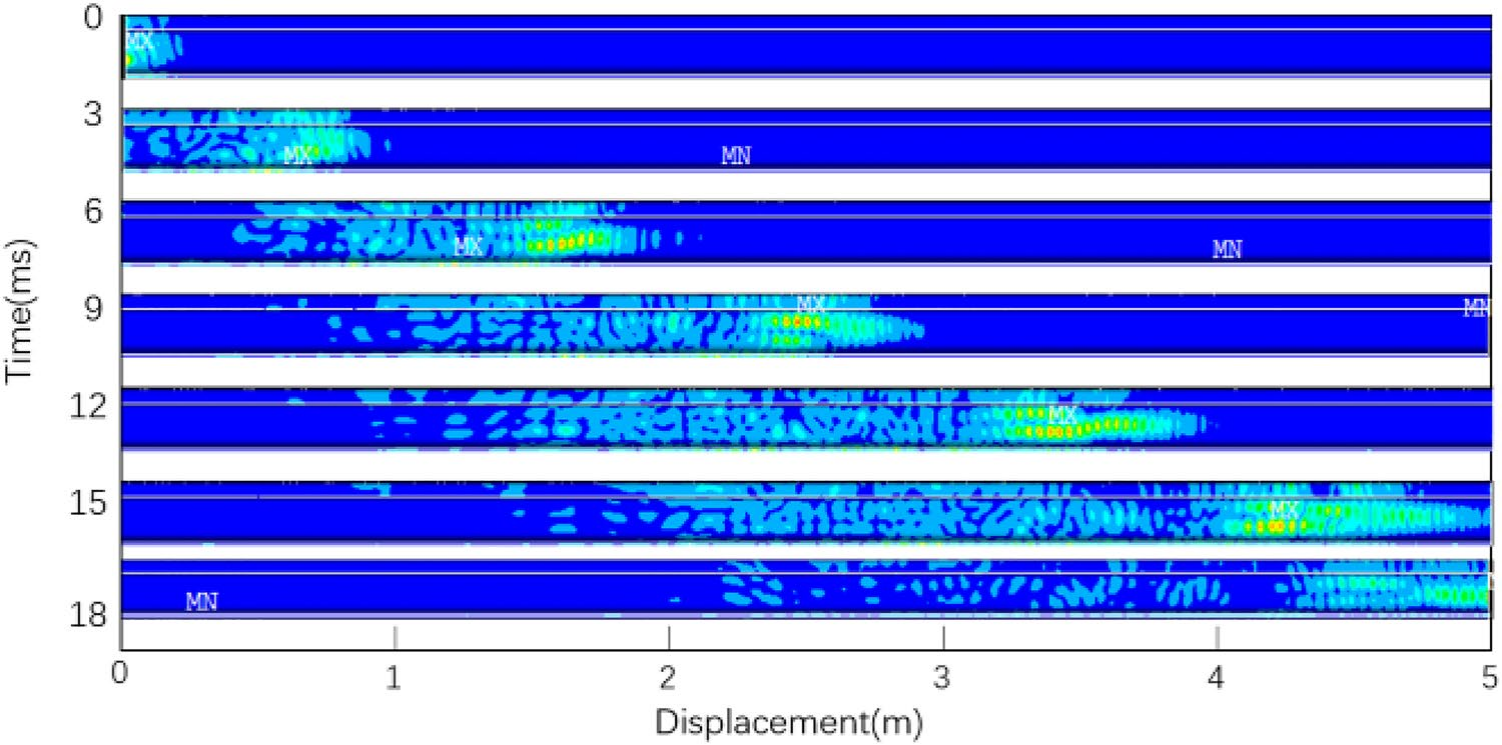
Deformation process of rail.

The deformation process of rail with time obtained by simulation is shown in Fig. 13.

**Figure 14:**
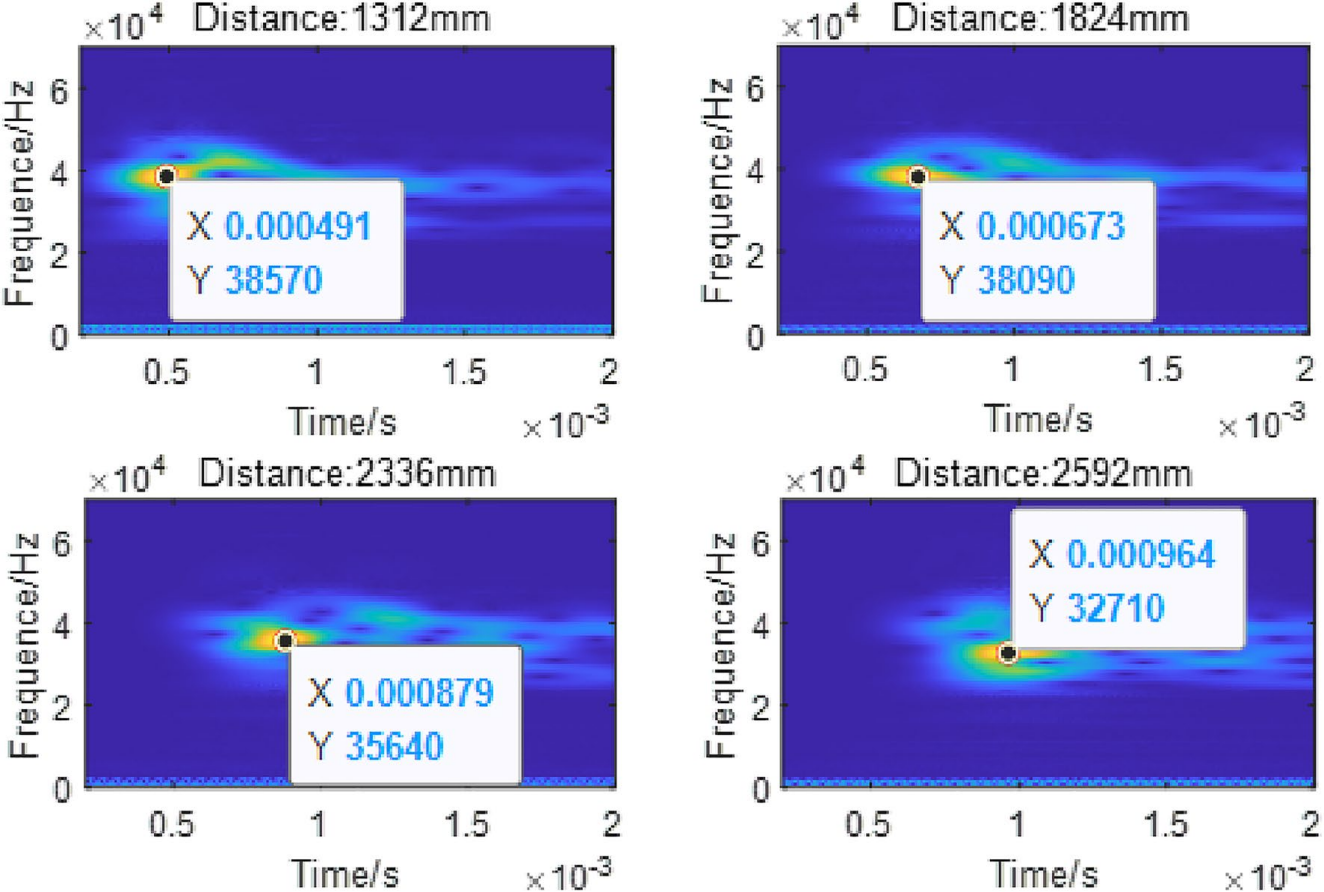
Wavelet transform diagram.

The vibration data of 240 nodes at the same horizontal position as the excitation point and within the range of 1184–3104 mm from the excitation point are extracted, and continuous wavelet transform is performed on the data to obtain the wavelet time–frequency diagram, as shown in Fig. 14.

The "distance" in the figure represents the distance from the receiving node to the excitation point. The point with the highest frequency component is marked in the figure. It can be seen from the figure that the frequency of the signal is concentrated near 35kHz, and the calculated speed of the guided wave energy concentration point reaching each node is shown in Table 2, which is very close to the group speed of 2669m/s of mode 9 calculated by the SAFE method. Therefore, the mode propagating in the rail is mainly mode 9.

**Table 2:** Group speed of the guided wave.

Distance (mm)	Time (us)	Group velocity (m/s)	Bias (%)
1312	491	2672.10	0.12
1824	673	2710.25	1.55
2336	879	2657.57	− 0.43
2592	964	2688.80	0.74

### Mode verification based on mode shapes

The Sect. 2080mm from the excitation point was selected as a specific cross-section for mode validation, named Ω. The theoretical group velocity of mode 9 solved by the SAFE method is 2669 m/s. According to the group velocity, it can be calculated that the time for mode 9 to reach the section Ω is about 0.000779s.

$$t = \frac{{2080\,{\text{mm}}}}{{2669\,{\text{m/s}}}} = 0.000779\,{\text{s}}$$

**Figure 15:**
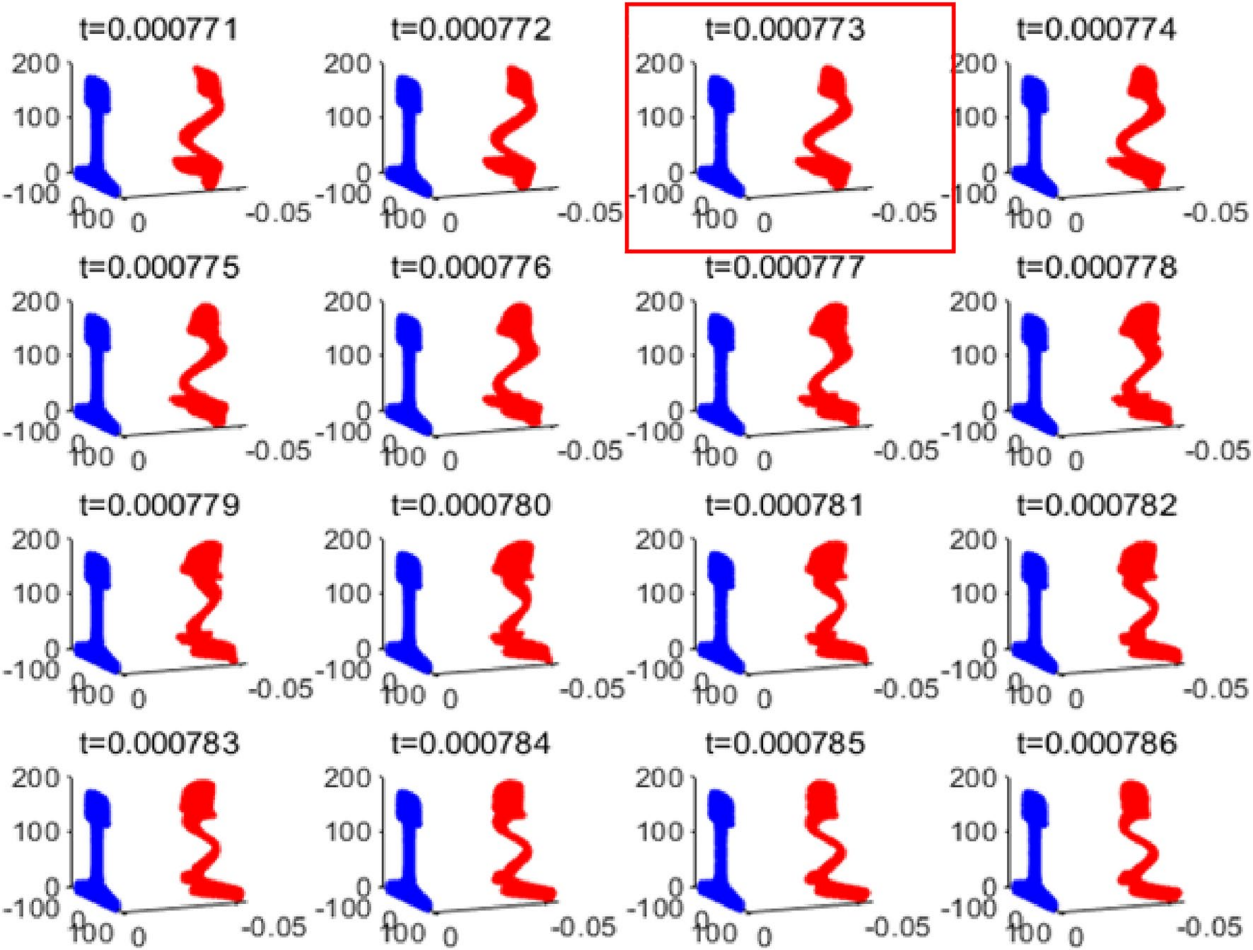
Vibration shapes diagram of section Ω near 0.000779 s.

The vibration shapes of section Ω near the time of T = 0.000779 s are drawn, as shown in Fig. 15. The vibration shape at t = 0.000773 is selected for analysis after comparison.

**Figure 16:**
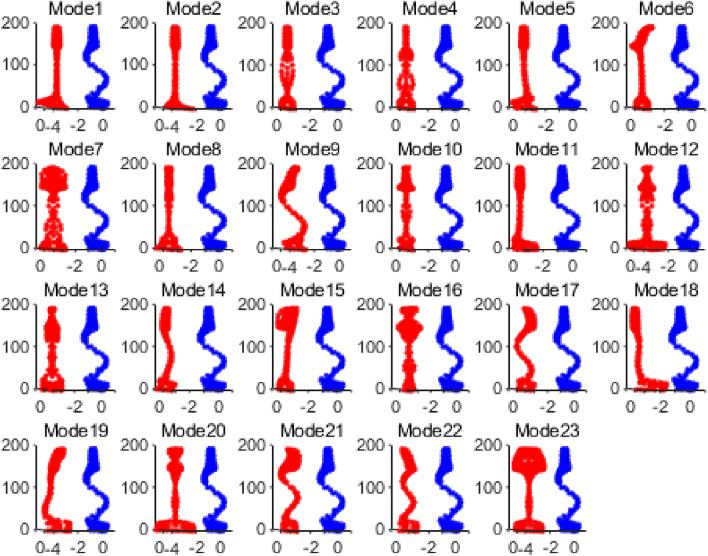
Comparison diagram of vibration shapes of section Ω and various modes at 0.000773 s.

The vibration shapes of 23 modes existing in the rail at 35kHz solved by the SAFE method are drawn and compared with the vibration shapes of section Ω at t = 0.000773s. The comparison diagram is shown in Fig. 16, with the vibration shape of section Ω at 0.000773 s in the right part of each subplot and the vibration shapes of each mode in the left part.

The 340 nodes of the section are numbered, and the vibration data of the section Ω at that time are extracted according to the three directions of *x*, *y*, and *z* and formed into the vector **q**^Ω^:

12 $$\begin{array}{*{20}c} {{\mathbf{q}}^{{\Omega }} = \left[ {\begin{array}{*{20}c} {u_{x1}^{{\Omega }} } & {u_{y1}^{{\Omega }} } & {\begin{array}{*{20}c} {u_{z1}^{{\Omega }} } & \cdots & {\begin{array}{*{20}c} {u_{xn}^{{\Omega }} } & {u_{yn}^{{\Omega }} } & {u_{zn}^{{\Omega }} } \\ \end{array} } \\ \end{array} } \\ \end{array} } \right]} \\ \end{array}$$

Similarly, the vibration data of the 23 modes are extracted in the *x*, *y*, and *z*-directions to form one-dimensional data and normalized to form the vector **q**^*m*^:

13 $$\begin{array}{*{20}c} {{\mathbf{q}}^{m} = \left[ {\begin{array}{*{20}c} {u_{x1}^{m} } & {u_{y1}^{m} } & {\begin{array}{*{20}c} {u_{z1}^{m} } & \cdots & {\begin{array}{*{20}c} {u_{xn}^{m} } & {u_{yn}^{m} } & {u_{zn}^{m} } \\ \end{array} } \\ \end{array} } \\ \end{array} } \right]} \\ \end{array}$$

The difference in vibration between the section Ω and the mode *m* is expressed in terms of the Manhattan distance, i.e.

14 $$D\left( {\Omega ,m} \right) = \begin{array}{*{20}c} {\mathop \sum \limits_{i = 1}^{n} \left( {\left| {u_{xi}^{m} - u_{xi}^{\Omega } } \right| + \left| {u_{yi}^{m} - u_{yi}^{\Omega } } \right| + \left| {u_{zi}^{m} - u_{zi}^{\Omega } } \right|} \right)} \\ \end{array}$$

A difference value is obtained for each mode, and the 23 difference values are sorted in ascending order to get the results shown in Table 3.

**Table 3:** Difference value (D-value) between section Ω and the vibration shape of each mode at 0.000773 s.

Mode	9	2	1	5
D-value	256.1907	263.3642	266.8521	270.5071
Mode	3	8	14	15
D-value	273.2616	288.3123	323.6595	338.7663
Mode	4	18	11	19
D-value	347.0474	368.7845	371.1023	377.0815
Mode	23	13	22	6
D-value	388.8818	392.8972	396.9228	399.5571
Mode	12	7	10	21
D-value	403.0213	406.0222	412.2435	414.2943
Mode	17	20	16	
D-value	418.8620	425.0082	481.8620	

**Figure 17:**
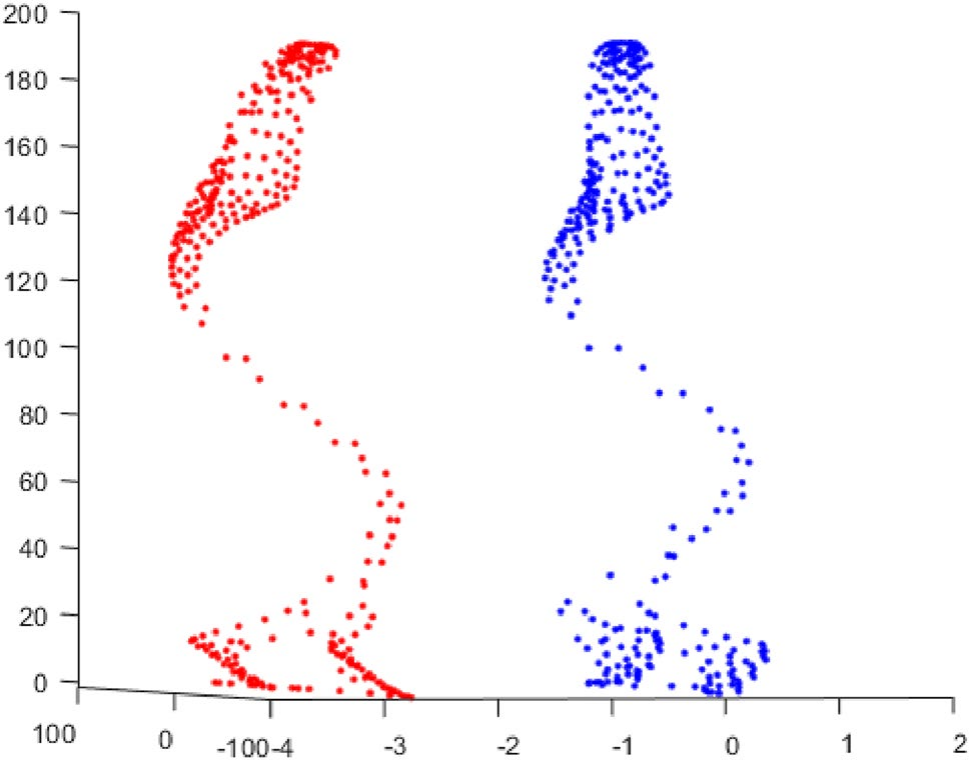
Comparison diagram of vibration shape of section Ω and mode 9 at 0.000773 s.

It can be seen from the table that when t = 0.000773, the difference between the vibration of section Ω and mode 9 is the smallest; that is, the vibration shape of section Ω is most similar to that of mode 9. Figure 17 shows the comparison between the vibration of mode 9 and the vibration of section Ω at the moment 0.000773s.

By comparing the group velocity and vibration shape diagrams, it can be demonstrated that the guided wave mode shown in the mode 9 vibration shape can be excited in the 75 kg/m rail by applying a five-cycle sinusoidal signal modulated by the Hanning window along the longitudinal rail direction at the node position (*x* = 10.4 mm, *y* = 88 mm).

## Simulation analysis of rail defect detection

**Figure 18:**
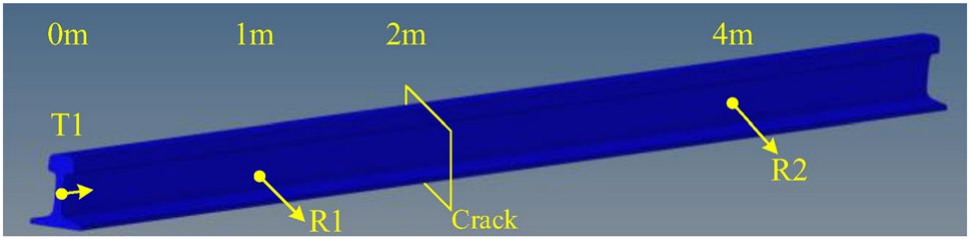
Exciting, receiving, and defect points for ANSYS simulation.

To further verify whether the mode excited at the node (*x* = 10.4mm, *y* = 88mm) has good sensitivity to the defects at the rail’s head, web, and foot, finite element simulation experiments were conducted (Supplementary Information). The exciting, receiving, and defect positions for the finite element simulation are shown in Fig. 18.

The guided wave excitation point T1 is located at the rail waist of the rail end, and the receiving point R1 is set 1m away from the excitation point to receive the echo signal generated by the guided wave at the defect. At a distance of 2m from the T1 point, the rail head, web, and foot defects are set, respectively. A receiving point R2 is set at 4m to receive the transmitted wave signal after the guided wave passes through the defect.

The five-cycle sinusoidal signal modulated by the Hanning window is applied at the rail web with a center frequency of 35 kHz. The simulation steps are shown in Table 4; four simulations were conducted, the first simulation was performed on the intact rail, and the remaining three were conducted with defects at the rail head, rail web, and rail foot, respectively.

**Table 4:** Defect settings of simulation experiment.

Number	Defect position	Defect size (mm)
1	No defect	0
2	Rail head	50 × 18 × 8
3	Rail web	10 × 50 × 8
4	Rail foot	50 × 18 × 8

**Figure 19:**
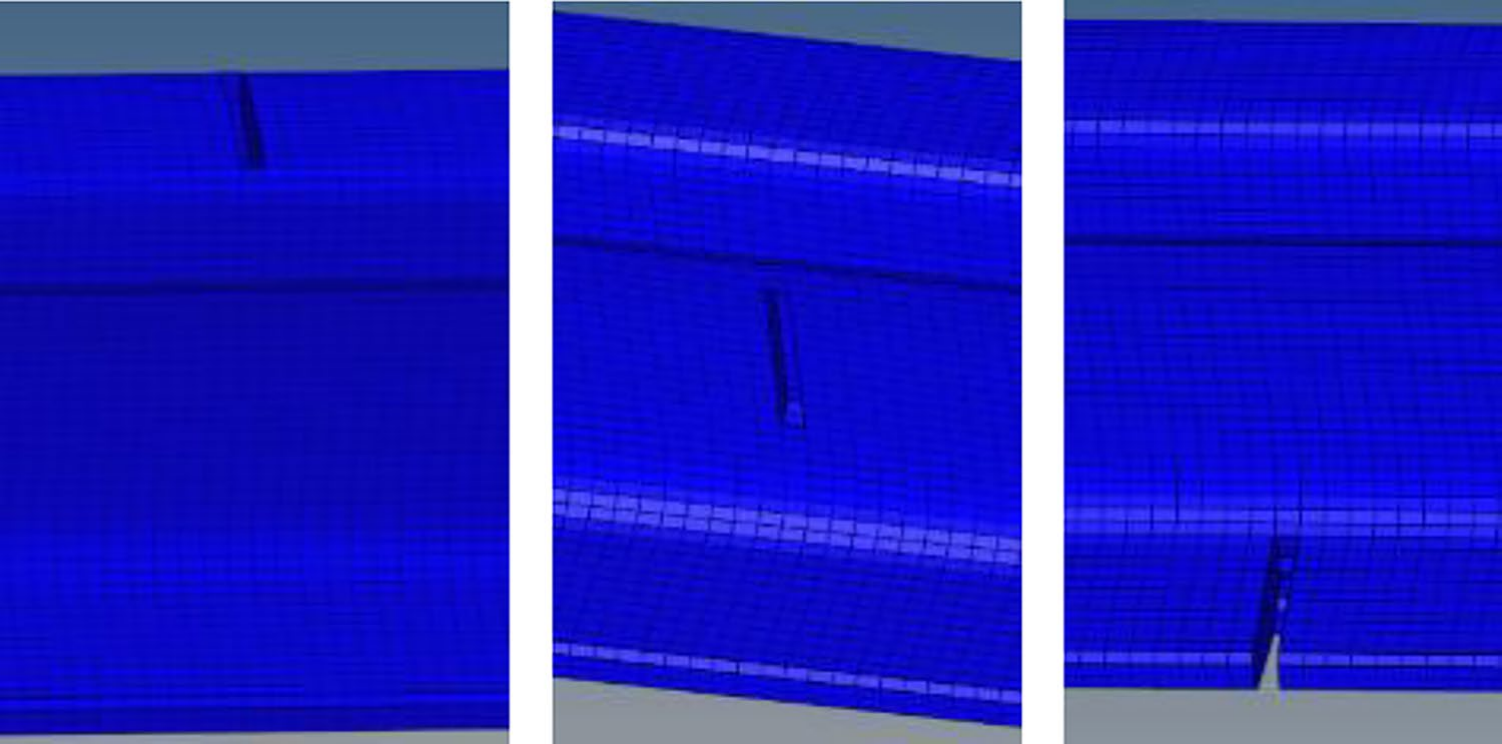
Settings of rail cracks at different positions.

The crack settings are shown in Fig. 19.

Four groups of signals are collected at the R1 point, the first group is the signal collected on the intact rail, and the second to fourth groups are echo signals of T1 excited guided wave signals after encountering defects at the rail head, rail web, and rail foot. By comparing the difference between the signals of the second to fourth groups and the signals of the first group, the sensitivity of the guided wave excited at the T1 position to the defects in different places was analyzed.

**Figure 20:**
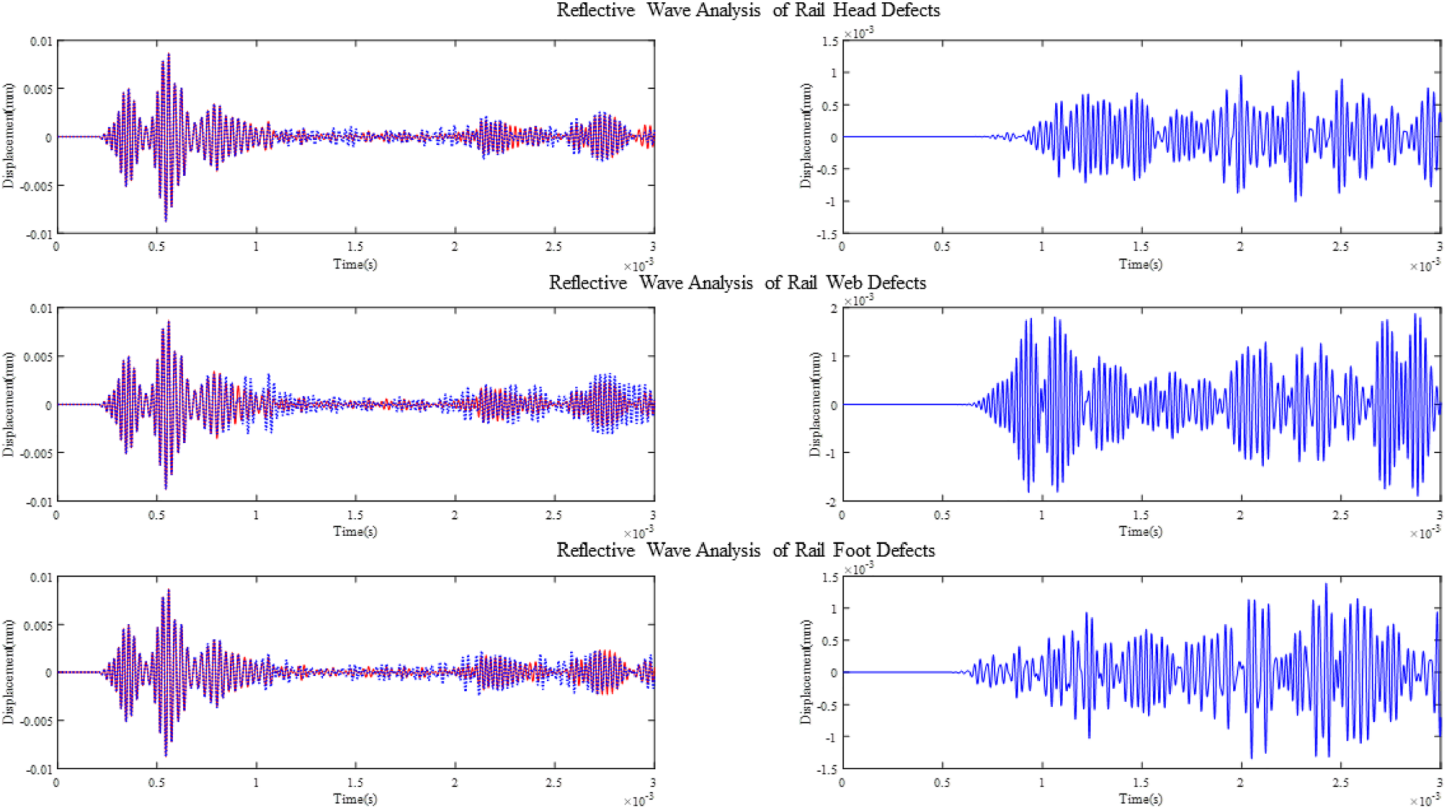
Comparison and differential analysis of reflected waves from defect and no defect at different positions.

The left plot in Fig. 20 shows the crack reflection signals received by R1 when the defect is at the rail head, rail web and rail foot, respectively, indicated by the red line. It is compared with the reflected wave at the corresponding position when there is no defect. The right side shows the result of the difference made between the two sets of signals.

It can be seen that the guided wave signals excited at point T1 show clear echo signals at the locations of cracks on the rail head, web, and foot.

Four groups of signals are collected at the R2 point, the first group is the signal collected on the intact rail, and the 2nd–4th groups are the transmitted waves of the guided wave signal excited at T1 after passing through the defects at the rail head rail web and rail foot, respectively. Comparing the signal attenuation of groups 2–4 with group 1 of the T1-excited guided wave signal after passing through the defects at different locations is analyzed.

**Figure 21:**
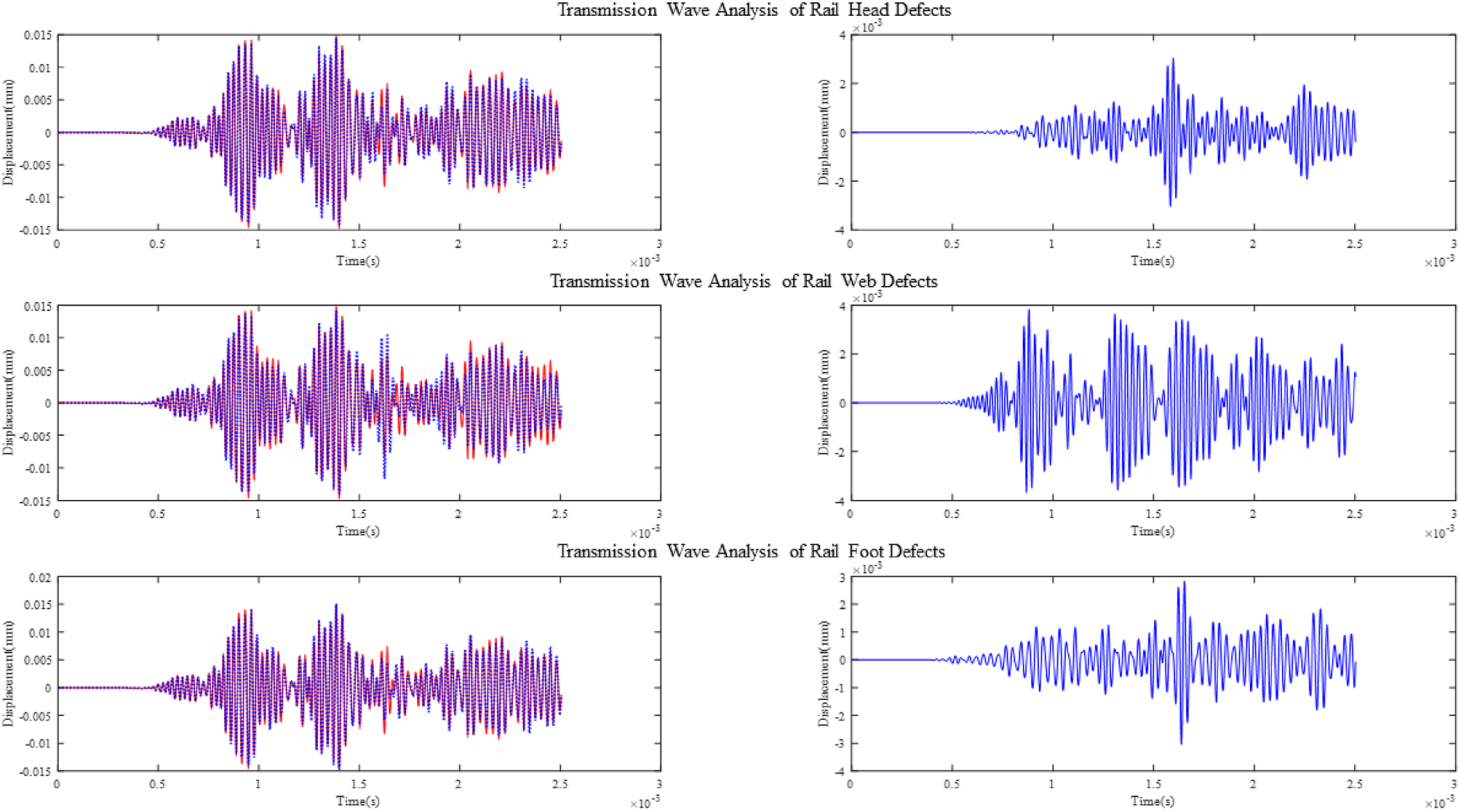
Comparison and differential analysis of transmitted waves from defect and no defect at different positions.

The left plot in Fig. 21 shows the crack transmission signals received by R2 when the defect is at the rail head, rail web and rail foot, respectively, indicated by the red line. It is also compared with the transmitted wave at the corresponding position when there is no defect. The right side shows the result of the difference made between the two sets of signals.

Based on the signal analysis from Fig. 20, the selected modes exhibit reflection coefficients of 0.105, 0.21, and 0.15 for defects at the rail head, rail web, and rail foot, respectively. This indicates that the chosen modes are sensitive to defects at these locations. Additionally, it can be observed from the figures that the strength of the defect echoes follows the order of rail web, rail foot, and rail head, consistent with the energy analysis shown in Fig. 5, further confirming that the excited mode primarily corresponds to Mode 9.

From Fig. 21, it can be observed that the selected modes exhibit different degrees of attenuation when passing through defects at different positions. Considering both the reflection and attenuation signals, by applying the excitation signal at the node positions x = 10.4mm and y = 88mm ,it is possible to detect significant cracks in the rail by analyzing the echo of the defect or analyzing the attenuation of the transmitted wave..

## Experimental results

**Figure 22:**
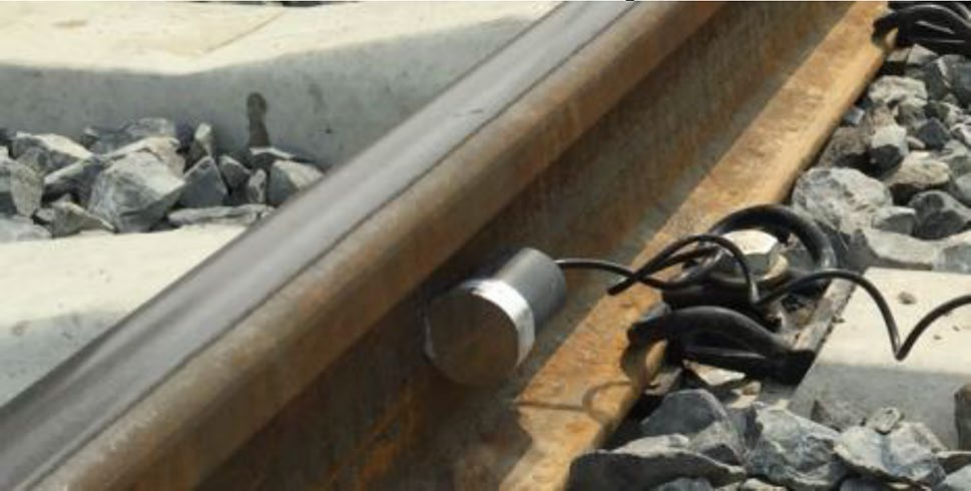
Transducer installation diagram.

Field experiments are conducted to further validate the effectiveness of the excitation mode applied to the actual rail line. The installation of the sandwich piezoelectric transducers in the field is illustrated in Fig. 22.

**Figure 23:**
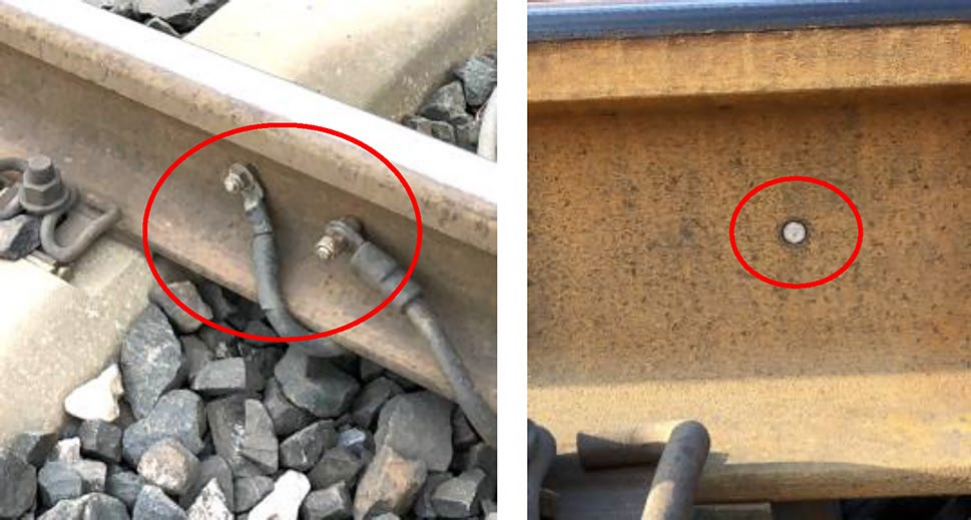
Grounding holes for simulating defects.

Firstly, the defect detection experiment was carried out. It is impossible to make holes or cut out other defects on the actual operating line. Therefore, the preset earthing hole on the line was taken as the defect point. The line earthing holes are shown in Fig. 23.

**Figure 24:**
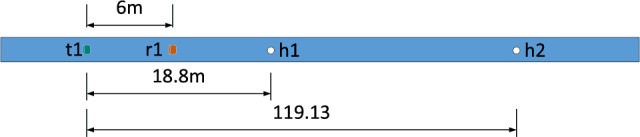
Experimental line setup.

The experimental setup is shown in Fig. 24. The exciting transducer is installed at point t1, as shown in Fig. 22. The receiving transducer r1 is 6m away from the exciting transducer, the first earthing hole h1 is 18.8m away from the exciting transducer, and the second grounding hole h2 is 119.13m away from the exciting transducer.

**Figure 25:**
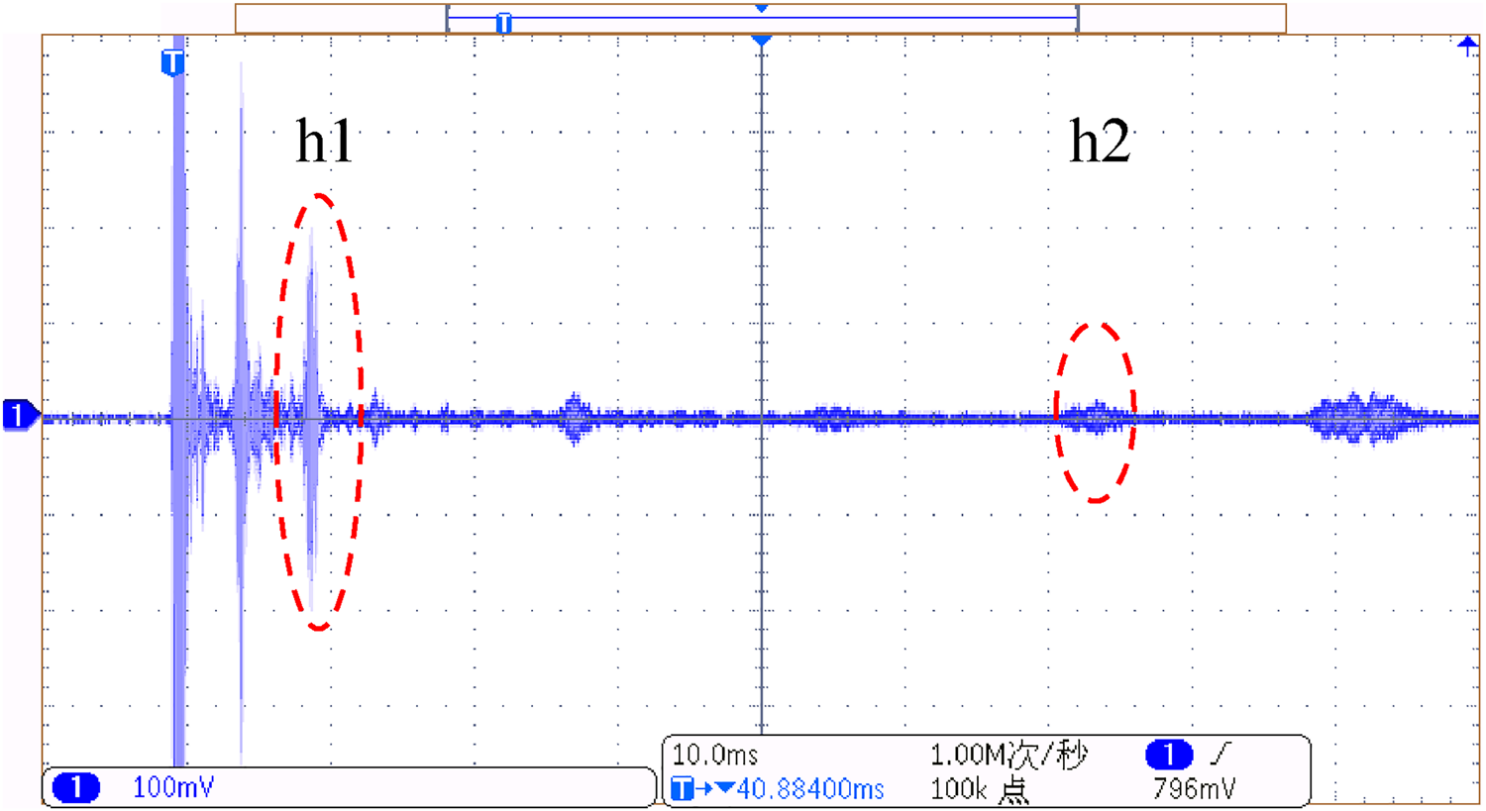
Field signal waveform.

The field signal collected by the oscilloscope is shown in Fig. 25. The echo signals of defects h1 and h2 can be observed.

To verify the long-distance propagation performance of guided wave modes excited at the rail web, a 1km attenuation experiment was carried out on the Beijing Circular Railway (Supplementary Information). The transducer was installed at the rail web, and the excitation signal shown in Fig. 12 was applied along the longitudinal direction of the rail.

To verify the long-distance propagation performance of guided wave modes excited at the rail web, an attenuation experiment was conducted on the Beijing Circular Railway with a radius of 1.5 km. The transducer was installed at the rail web, and the excitation signal shown in Fig. 12 was applied along the longitudinal direction of the rail.

In the experiment, the amplitude of the excitation signal is ± 125V, and a receiving point is set at every 50m. We continued to use the excitation signal as shown in Fig. 12, exciting at a frequency of 35 kHz, and made efforts to ensure that the excitation point was the same as the selected excitation point in the simulation experiments. At each receiving point, three groups of data are measured, and the signal strength of the receiving point is determined by calculating the average peak energy value across the three groups of data. The data are shown in Table 5. Through experimental measurement, the amplitude of the guided wave signal can still reach more than 4.7mV after 1km propagation.

**Table 5:** Data from the field experiments.

Distance (m)	Energy (V^2^)
50	14.23606
100	8.190338
150	7.03298
200	3.220529
250	3.274978
300	2.600834
350	1.776809
400	1.079978
450	0.532596
500	0.372291
550	0.040284
600	0.003655
650	0.003576
700	0.002464
750	0.001573
800	0.001172
850	0.000266
900	5.15e − 05
950	6.11e − 05
1000	2.24e − 05

**Figure 26:**
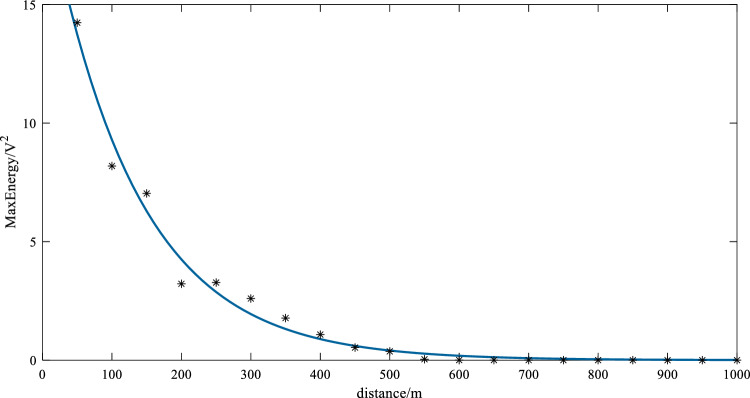
Attenuation curve on the Beijing Circular Railway.

The data in Table 5 shows that the attenuation curve is fitted exponentially. The fitting effect is shown in Fig. 26, and the energy attenuation coefficient is calculated to be about 0.0078.

The attenuation experiment shows that the mode selected in this study can receive an effective signal at 1km when the excitation signal amplitude is ± 125V, indicating that this mode can be used for long-distance rail flaw detection.

**Figure 27:**
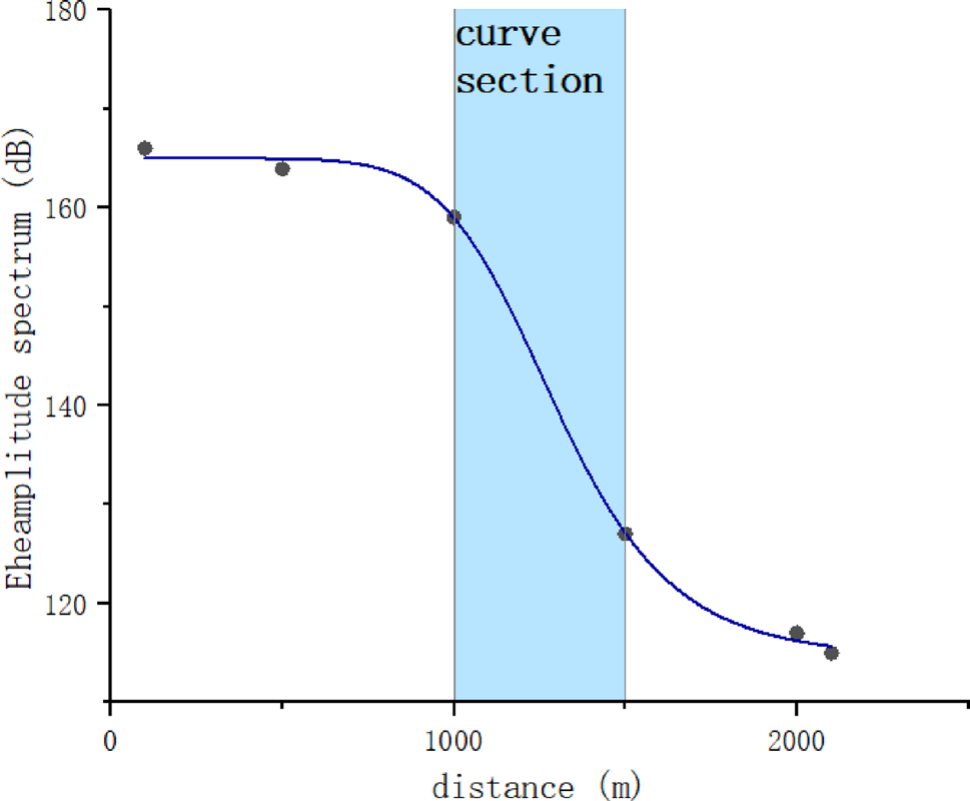
Attenuation curve with curve section.

To explore the influence of environmental factors on the attenuation of guided wave signals, we conducted additional experiments on two different railway tracks. One experiment was conducted on a straight track, while the other was conducted on a track with smaller radius curves. In these experiments, we used higher-precision three-axis accelerometer sensors and increased the excitation voltage to ± 300V. After excitation, signals were collected at different distances. The experimental data on a straight track were shown in Table 6 and the attenuation curves of the second experiment were plotted as shown in Fig. 27.

**Table 6:** Experimental data on a straight track.

Distance (m)	Eheamplitude spectrum (dB)
5	117.3
100	99.99
866	93.27
2500	68.53
3300	37.92

From Table 6, it can be observed that on the straight track segment with good operating conditions, the guided wave modes excited by this method exhibit smaller attenuation. Guided wave signals can still be detected at a distance of 3 km.

In Fig. 27, we can see that on curved track segments, the attenuation of guided wave signals increases. This indicates that the method proposed in this study is still influenced by environmental factors such as curves. To address this issue, we can increase sensor density in curved sections according to the actual condition of the track.

## Conclusion

In recent years, ultrasonic guided wave (UGW) has become an essential tool for non-destructive testing of railways. Aiming at the application of UGW in long-distance rail breakage monitoring of U78CrV rail, the main contributions of this paper are as follows.

1. The dispersion curve of UGW in the U78CrV rail is solved by the semi-analytical finite element method, and the guided wave mode suitable for monitoring the whole section monitoring of this rail is selected. The excitation point and mode are determined by calculating the vibration energy of this mode on the rail surface.
2. The three-dimensional finite element simulation of ANSYS is used to analyze the rail vibration, and the group velocity of the vibration energy concentration point is solved by wavelet transform. It is verified that the selected mode is the primary mode existing in the rail.
3. A specific rail section is selected, the arrival time of the selected mode is calculated, and the vibration mode shape data near this time is extracted and compared with the mode shape of the semi-analytical mode to verify the successful excitation of the selected mode.
4. Through simulation and field experiment, it is verified that the guided wave mode excited in this paper is sensitive to the cracks at the rail head, rail web, and rail foot and can travel more than 1km in heavy haul railway rails. Remote online monitoring of rail defects or broken rails can be realized by detecting the reflected echo of defects or attenuating the guided wave signals after defects.

In conclusion, through experimental validation, the ultrasonic guided wave detection method proposed in this study achieves a detection distance of over 3 km on straight sections, demonstrating promising prospects in long-distance rail break detection. An important development direction for future rail integrity health monitoring is ultrasonic guided wave-based long-distance rail break detection. It involves selecting and exciting modes with minimal influence from track conditions and environmental factors and low attenuation rates by deeply studying the propagation mechanism of guided waves.

### Ethical approval

This article does not contain any studies with human participants or animals performed by any authors.

## Supplementary Information

Supplementary Information.
