# Supplementary figures and images for: Study on monitoring broken rails of heavy haul railway based on ultrasonic guided wave
Source: Sci Rep. 2024 Apr 15;14:8667. doi: 10.1038/s41598-024-59328-5 (PMC11385469; doi:10.1038/s41598-024-59328-5)

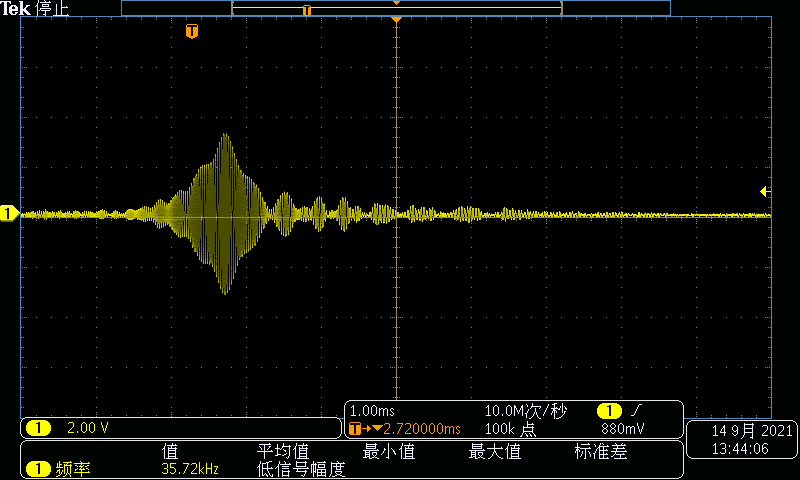

Supplement: Supplementary file 1 — Supplementary Information. [file 41598_2024_59328_MOESM1_ESM.zip › Decay experiment data/tek0000.png]

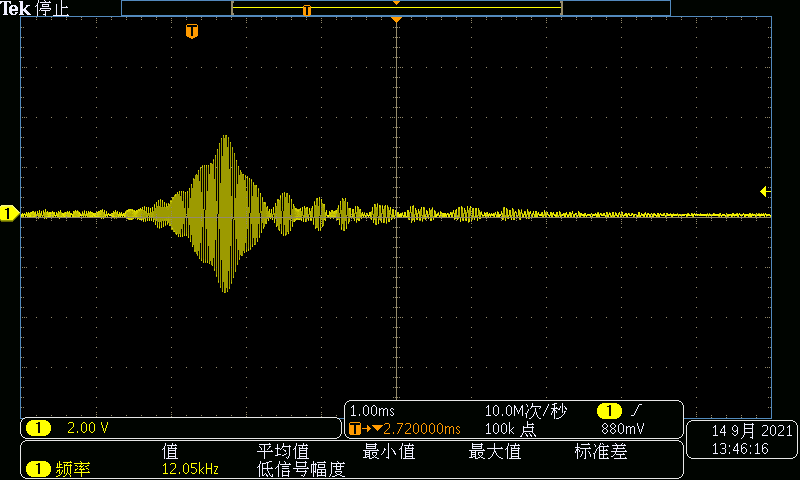

Supplement: Supplementary file 1 — Supplementary Information. [file 41598_2024_59328_MOESM1_ESM.zip › Decay experiment data/tek0001.png]

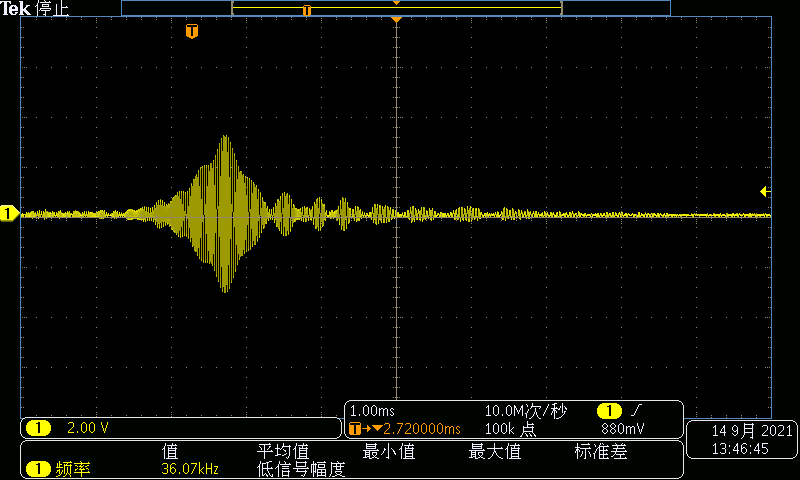

Supplement: Supplementary file 1 — Supplementary Information. [file 41598_2024_59328_MOESM1_ESM.zip › Decay experiment data/tek0002.png]

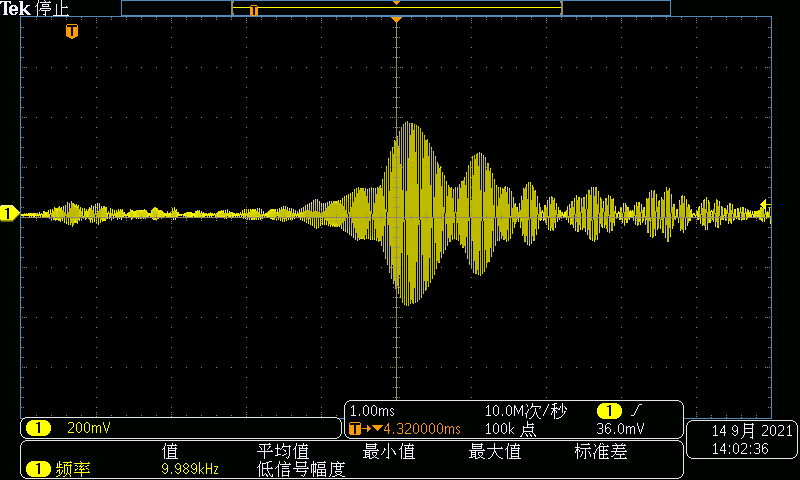

Supplement: Supplementary file 1 — Supplementary Information. [file 41598_2024_59328_MOESM1_ESM.zip › Decay experiment data/tek0003.png]

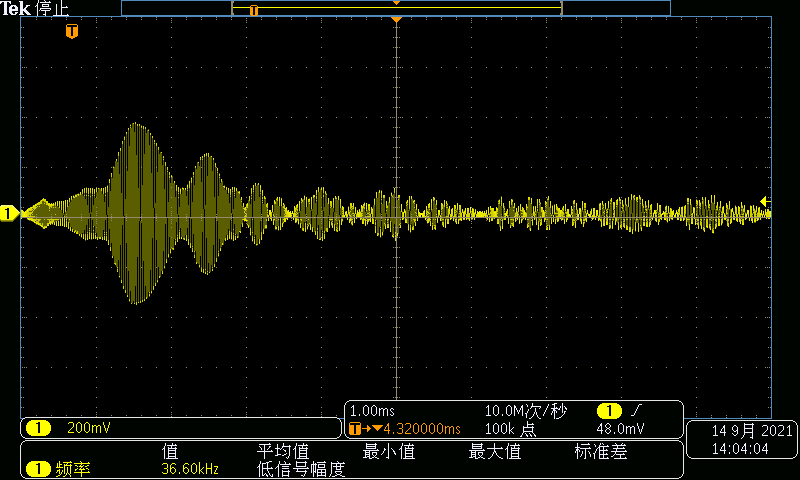

Supplement: Supplementary file 1 — Supplementary Information. [file 41598_2024_59328_MOESM1_ESM.zip › Decay experiment data/tek0004.png]

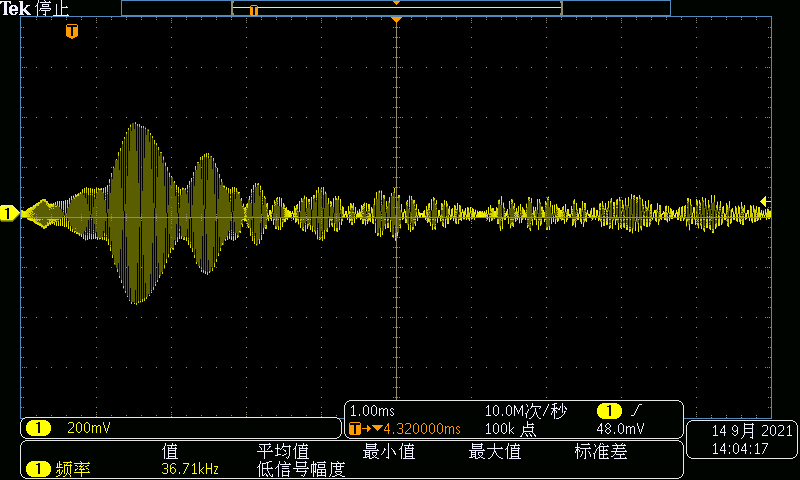

Supplement: Supplementary file 1 — Supplementary Information. [file 41598_2024_59328_MOESM1_ESM.zip › Decay experiment data/tek0005.png]

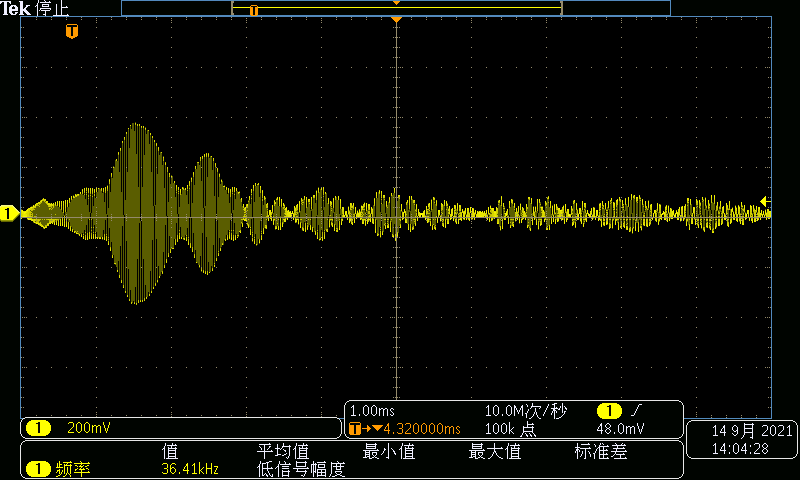

Supplement: Supplementary file 1 — Supplementary Information. [file 41598_2024_59328_MOESM1_ESM.zip › Decay experiment data/tek0006.png]

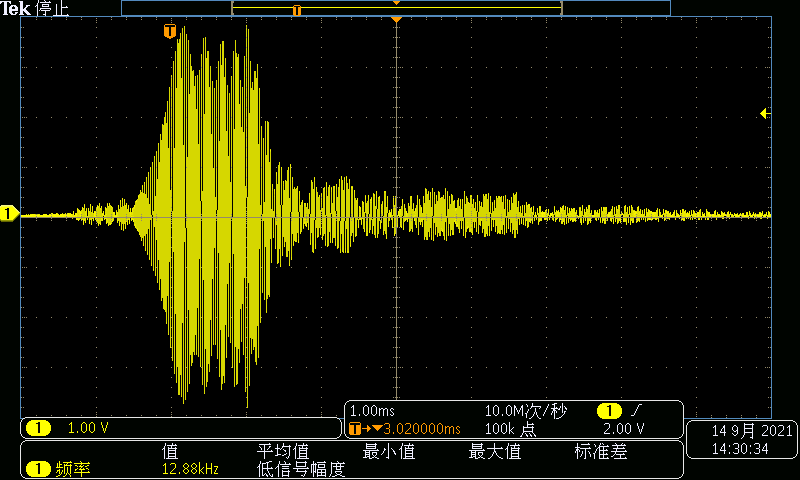

Supplement: Supplementary file 1 — Supplementary Information. [file 41598_2024_59328_MOESM1_ESM.zip › Decay experiment data/tek0007.png]

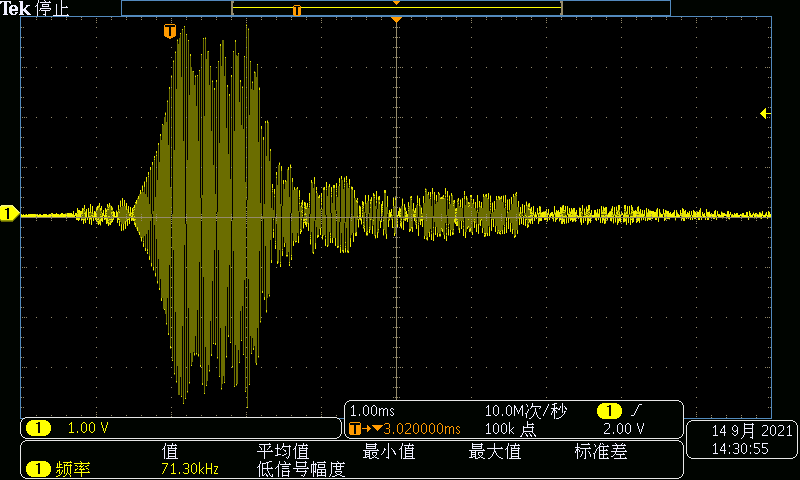

Supplement: Supplementary file 1 — Supplementary Information. [file 41598_2024_59328_MOESM1_ESM.zip › Decay experiment data/tek0008.png]

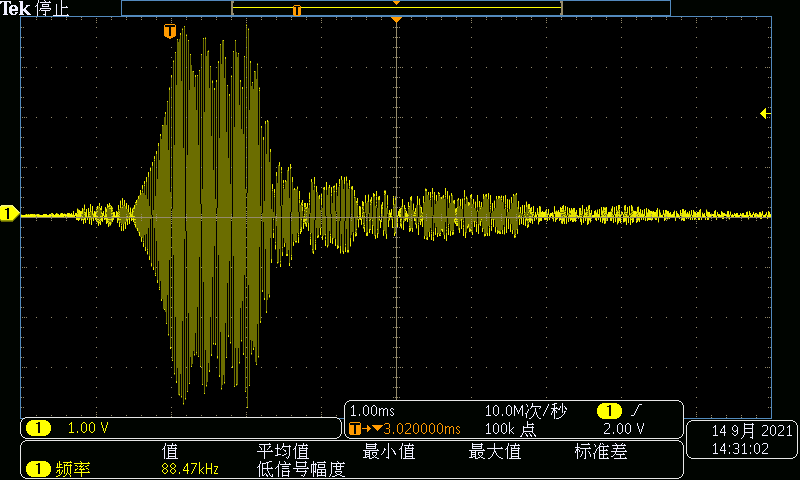

Supplement: Supplementary file 1 — Supplementary Information. [file 41598_2024_59328_MOESM1_ESM.zip › Decay experiment data/tek0009.png]

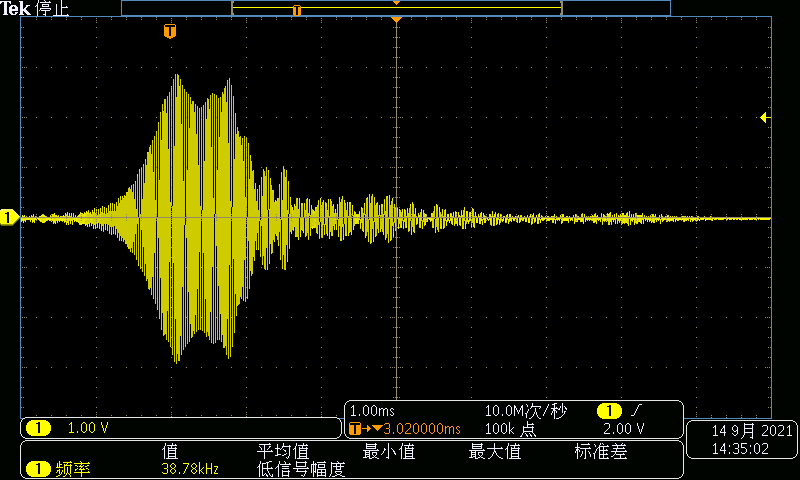

Supplement: Supplementary file 1 — Supplementary Information. [file 41598_2024_59328_MOESM1_ESM.zip › Decay experiment data/tek0010.png]

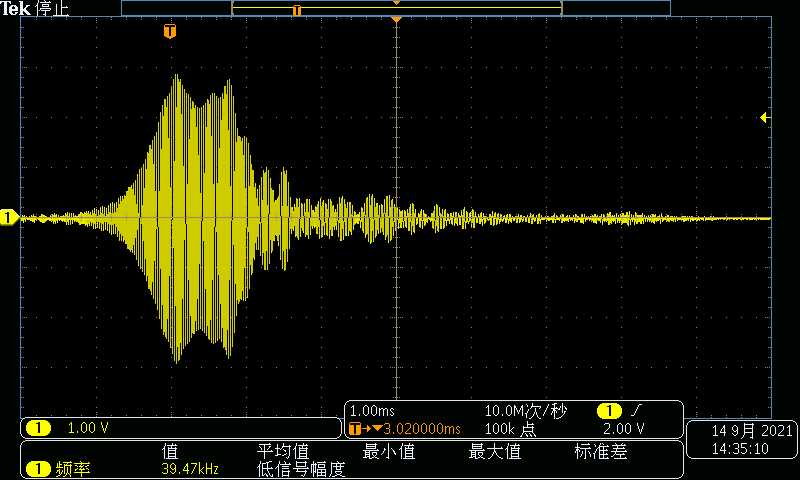

Supplement: Supplementary file 1 — Supplementary Information. [file 41598_2024_59328_MOESM1_ESM.zip › Decay experiment data/tek0011.png]

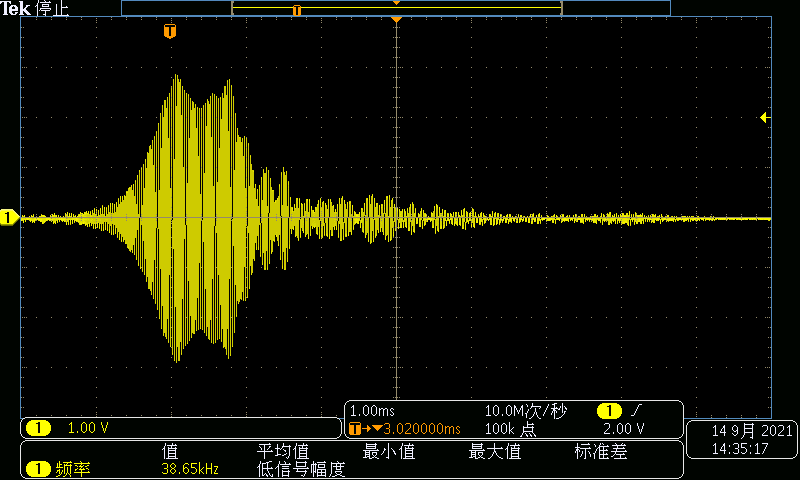

Supplement: Supplementary file 1 — Supplementary Information. [file 41598_2024_59328_MOESM1_ESM.zip › Decay experiment data/tek0012.png]

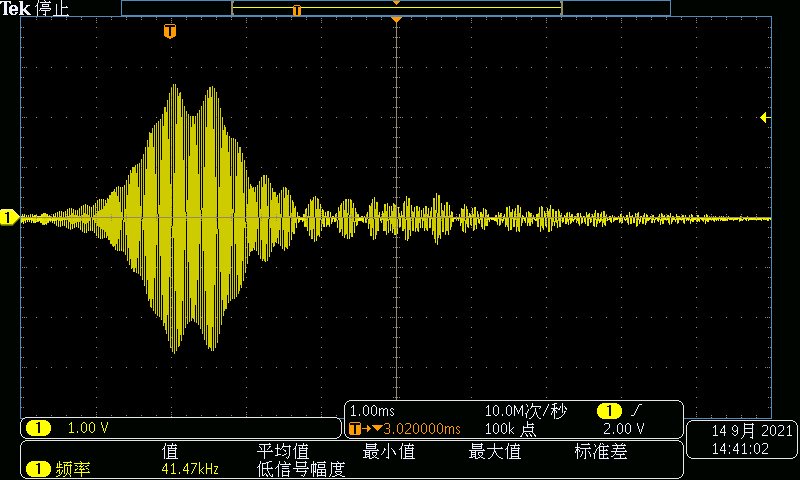

Supplement: Supplementary file 1 — Supplementary Information. [file 41598_2024_59328_MOESM1_ESM.zip › Decay experiment data/tek0013.png]

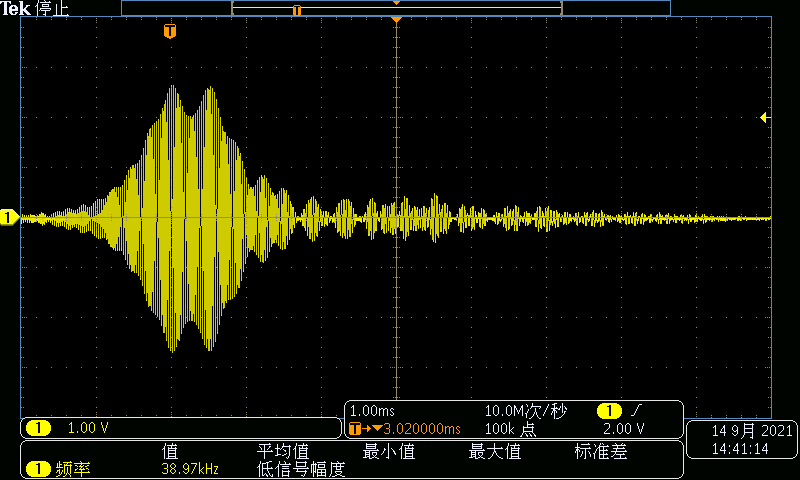

Supplement: Supplementary file 1 — Supplementary Information. [file 41598_2024_59328_MOESM1_ESM.zip › Decay experiment data/tek0014.png]

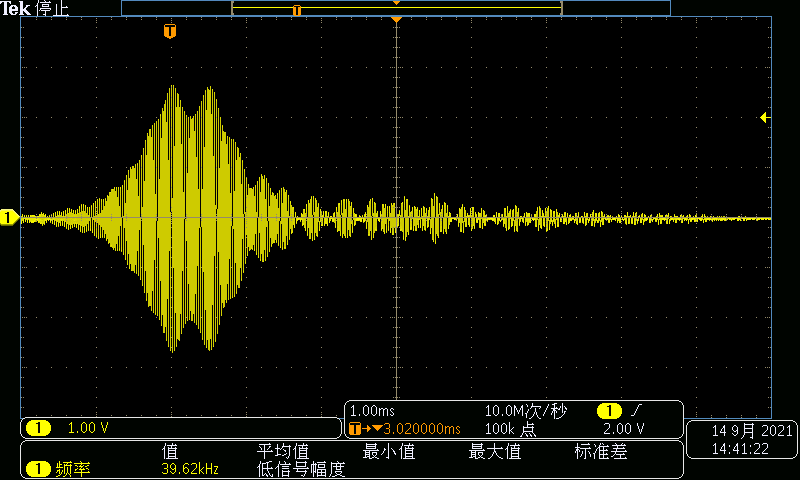

Supplement: Supplementary file 1 — Supplementary Information. [file 41598_2024_59328_MOESM1_ESM.zip › Decay experiment data/tek0015.png]

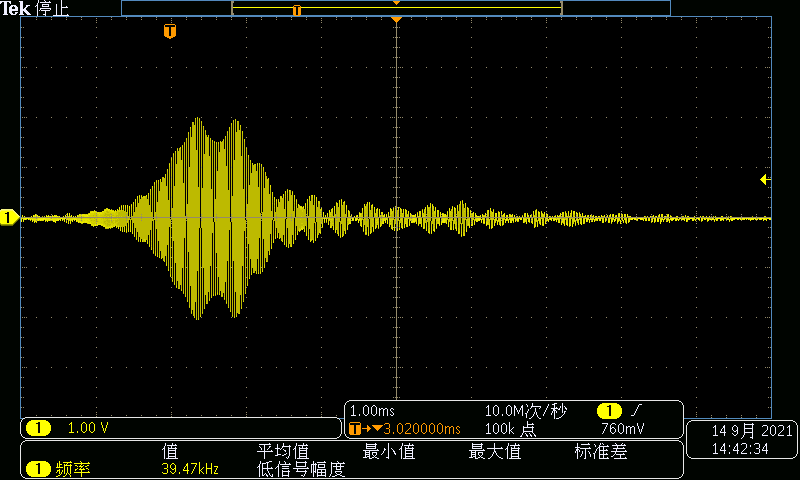

Supplement: Supplementary file 1 — Supplementary Information. [file 41598_2024_59328_MOESM1_ESM.zip › Decay experiment data/tek0016.png]

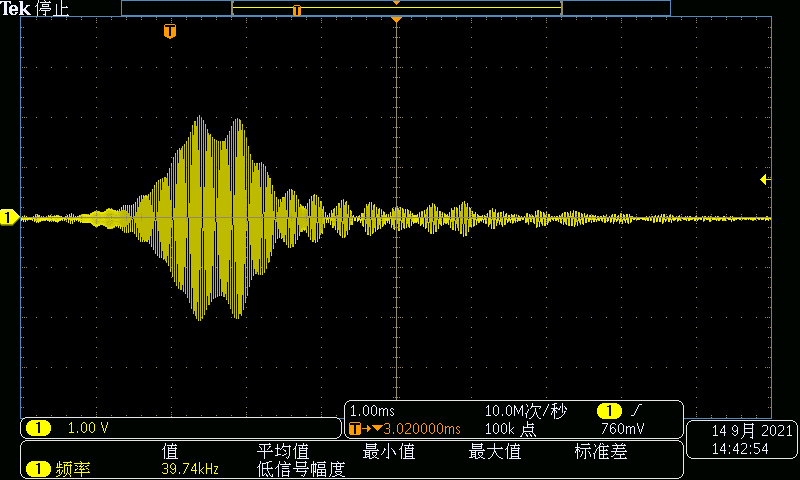

Supplement: Supplementary file 1 — Supplementary Information. [file 41598_2024_59328_MOESM1_ESM.zip › Decay experiment data/tek0017.png]

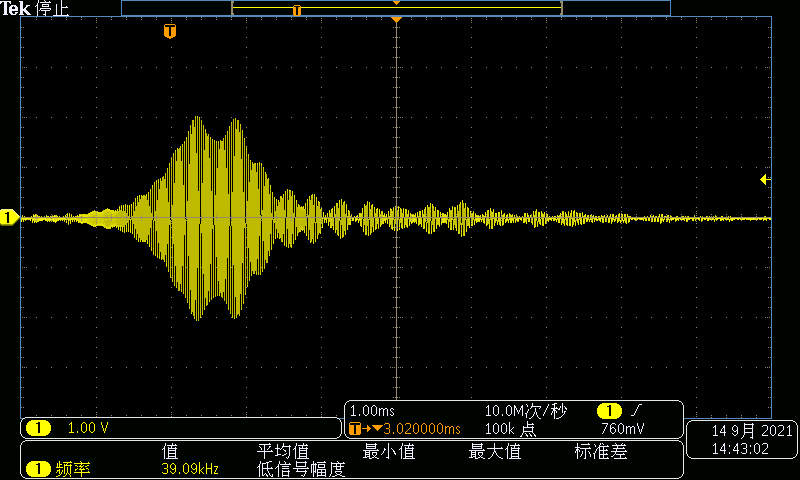

Supplement: Supplementary file 1 — Supplementary Information. [file 41598_2024_59328_MOESM1_ESM.zip › Decay experiment data/tek0018.png]

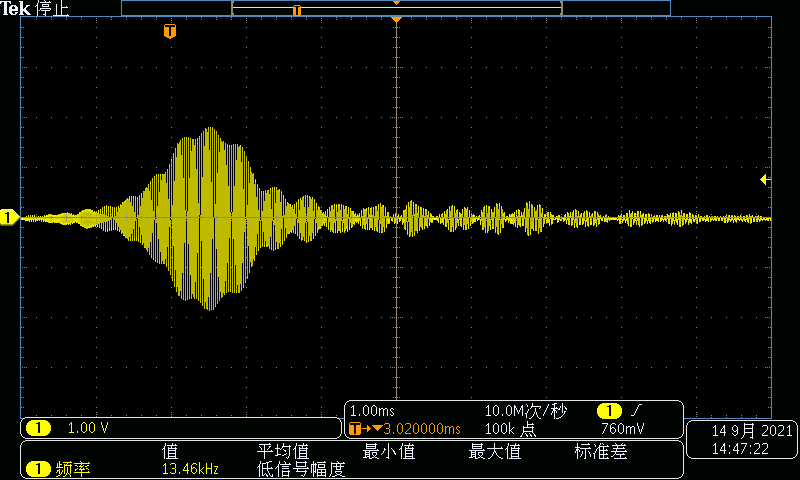

Supplement: Supplementary file 1 — Supplementary Information. [file 41598_2024_59328_MOESM1_ESM.zip › Decay experiment data/tek0019.png]

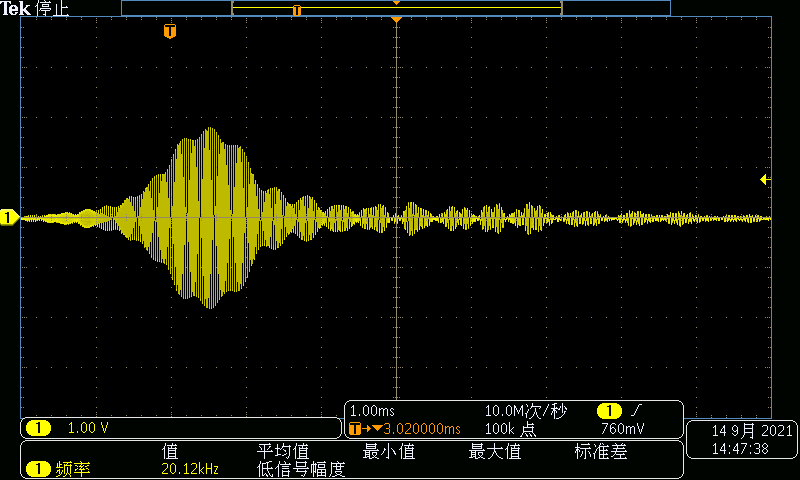

Supplement: Supplementary file 1 — Supplementary Information. [file 41598_2024_59328_MOESM1_ESM.zip › Decay experiment data/tek0020.png]

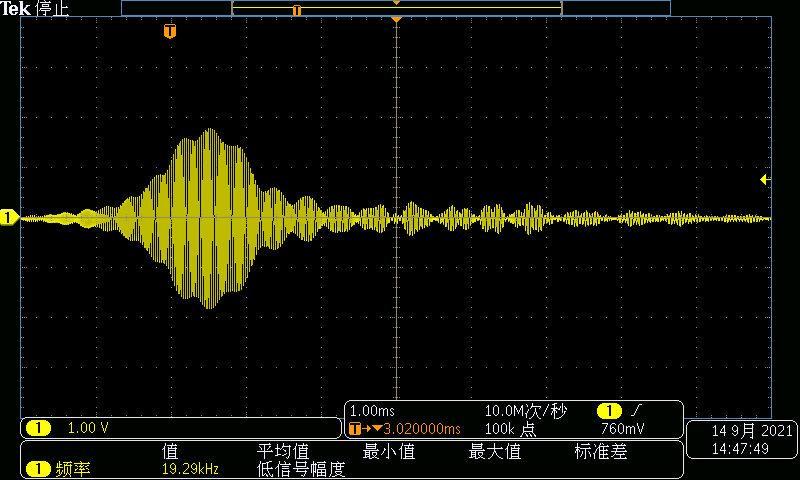

Supplement: Supplementary file 1 — Supplementary Information. [file 41598_2024_59328_MOESM1_ESM.zip › Decay experiment data/tek0021.png]

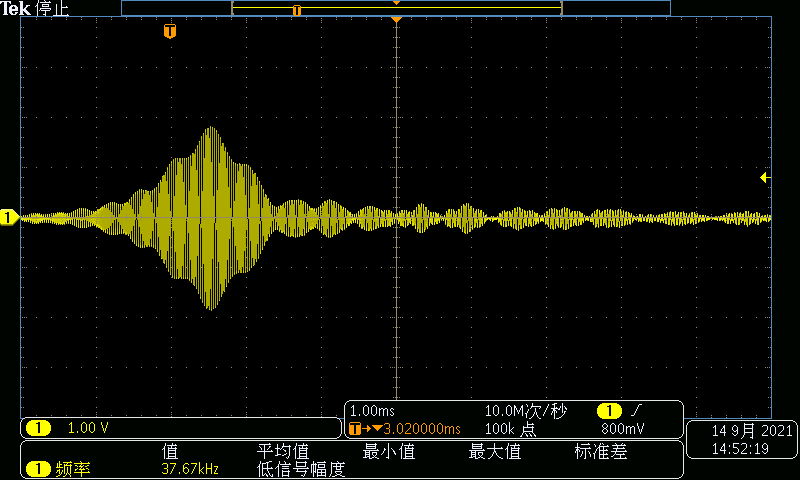

Supplement: Supplementary file 1 — Supplementary Information. [file 41598_2024_59328_MOESM1_ESM.zip › Decay experiment data/tek0022.png]

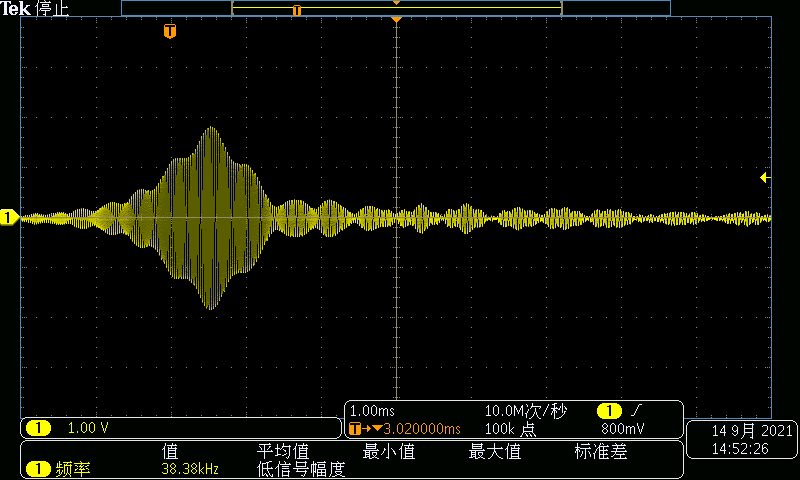

Supplement: Supplementary file 1 — Supplementary Information. [file 41598_2024_59328_MOESM1_ESM.zip › Decay experiment data/tek0023.png]

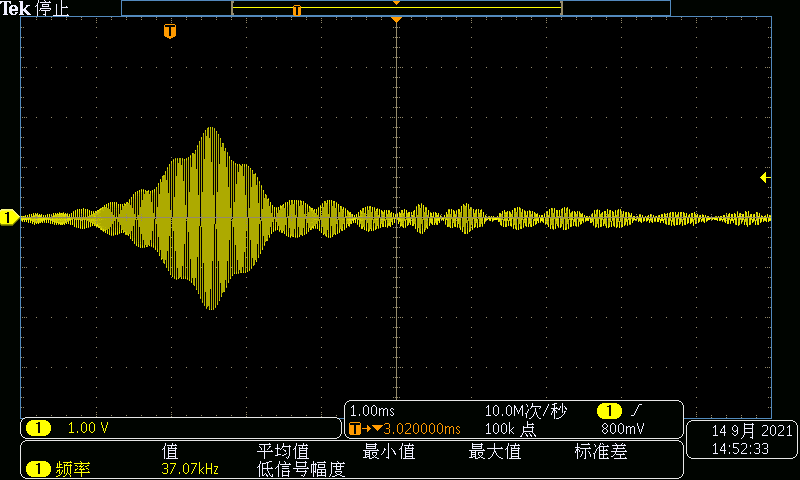

Supplement: Supplementary file 1 — Supplementary Information. [file 41598_2024_59328_MOESM1_ESM.zip › Decay experiment data/tek0024.png]

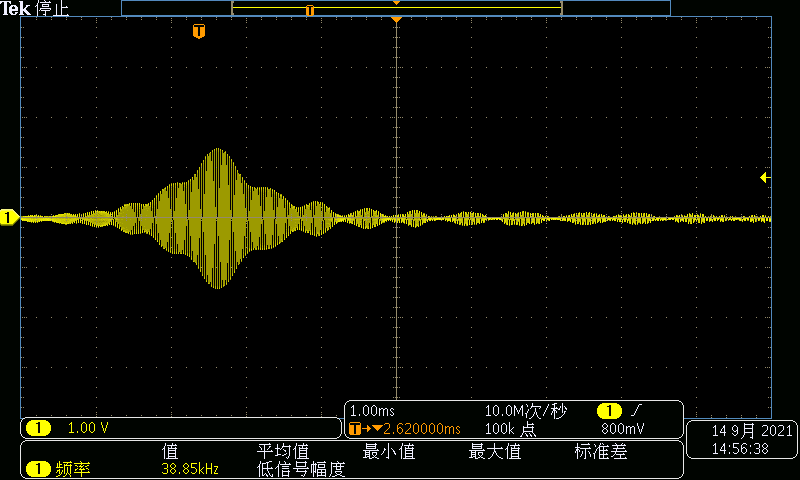

Supplement: Supplementary file 1 — Supplementary Information. [file 41598_2024_59328_MOESM1_ESM.zip › Decay experiment data/tek0025.png]

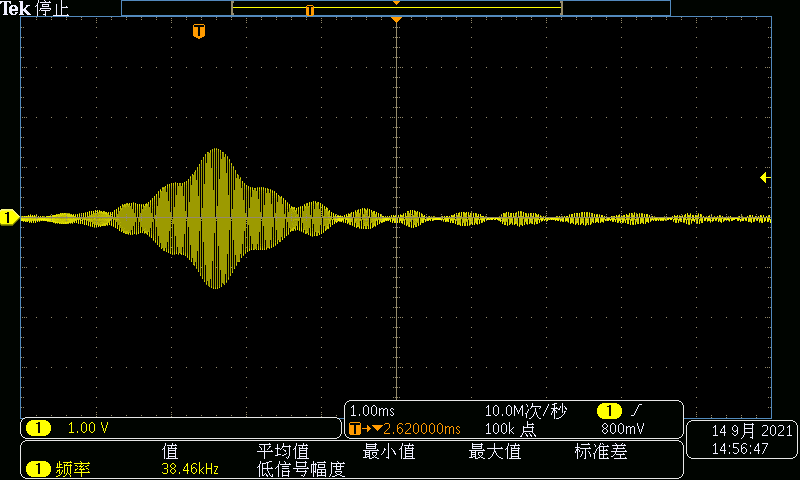

Supplement: Supplementary file 1 — Supplementary Information. [file 41598_2024_59328_MOESM1_ESM.zip › Decay experiment data/tek0026.png]

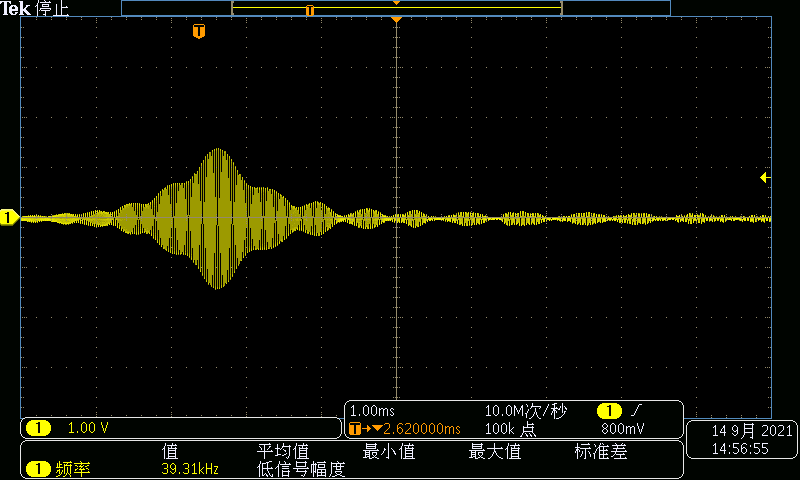

Supplement: Supplementary file 1 — Supplementary Information. [file 41598_2024_59328_MOESM1_ESM.zip › Decay experiment data/tek0027.png]

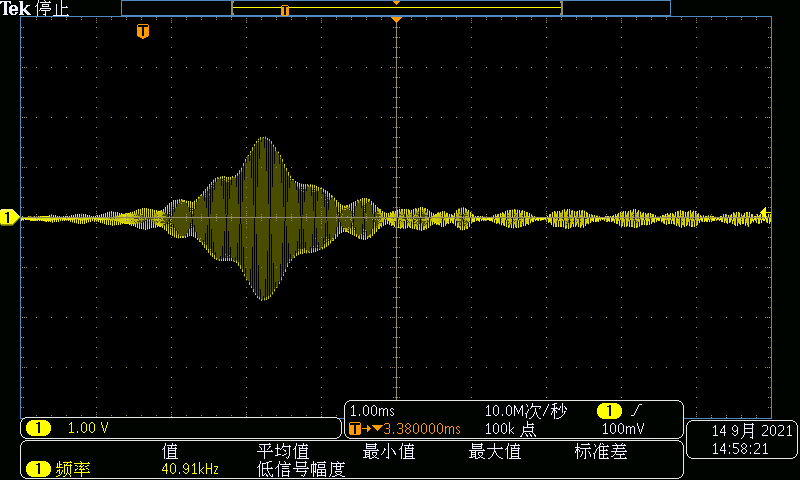

Supplement: Supplementary file 1 — Supplementary Information. [file 41598_2024_59328_MOESM1_ESM.zip › Decay experiment data/tek0028.png]

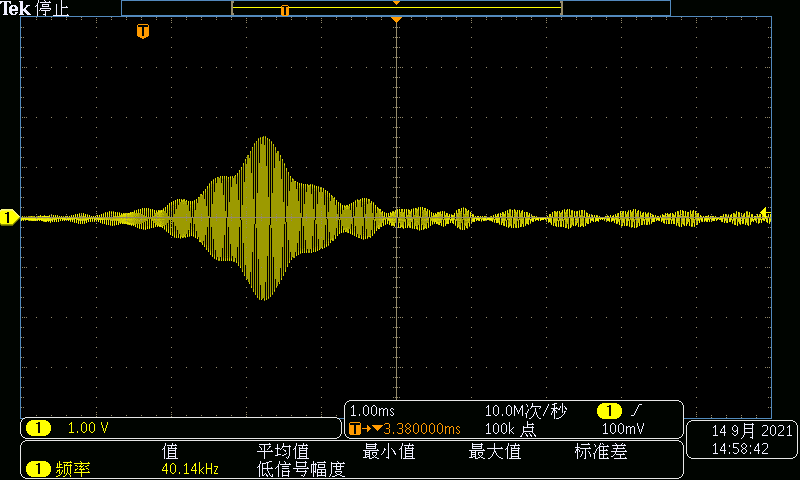

Supplement: Supplementary file 1 — Supplementary Information. [file 41598_2024_59328_MOESM1_ESM.zip › Decay experiment data/tek0029.png]

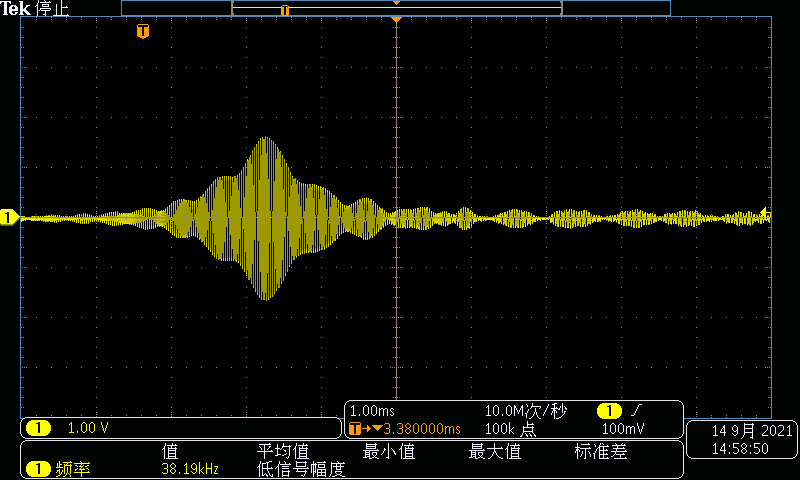

Supplement: Supplementary file 1 — Supplementary Information. [file 41598_2024_59328_MOESM1_ESM.zip › Decay experiment data/tek0030.png]

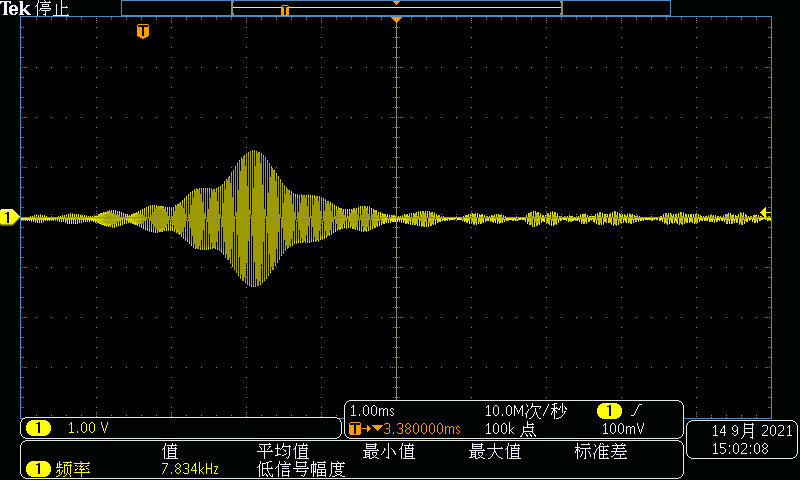

Supplement: Supplementary file 1 — Supplementary Information. [file 41598_2024_59328_MOESM1_ESM.zip › Decay experiment data/tek0031.png]

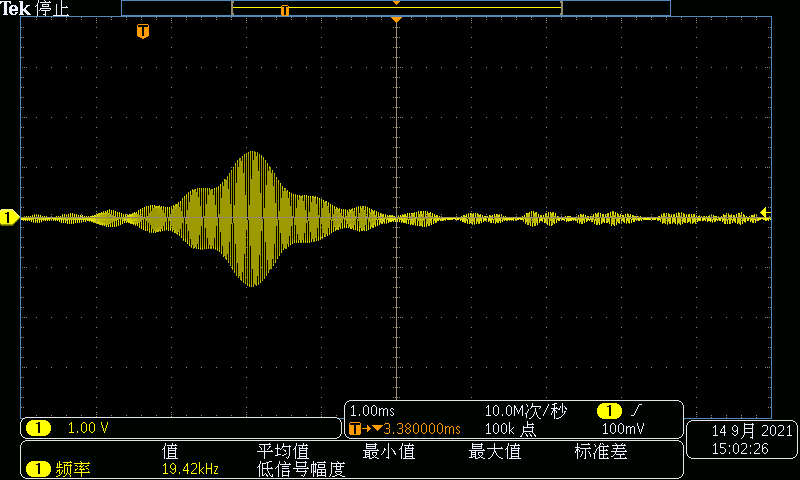

Supplement: Supplementary file 1 — Supplementary Information. [file 41598_2024_59328_MOESM1_ESM.zip › Decay experiment data/tek0032.png]

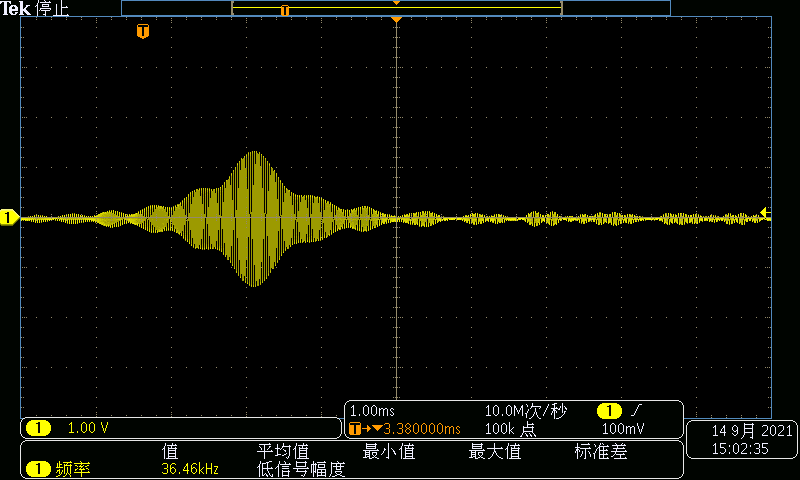

Supplement: Supplementary file 1 — Supplementary Information. [file 41598_2024_59328_MOESM1_ESM.zip › Decay experiment data/tek0033.png]

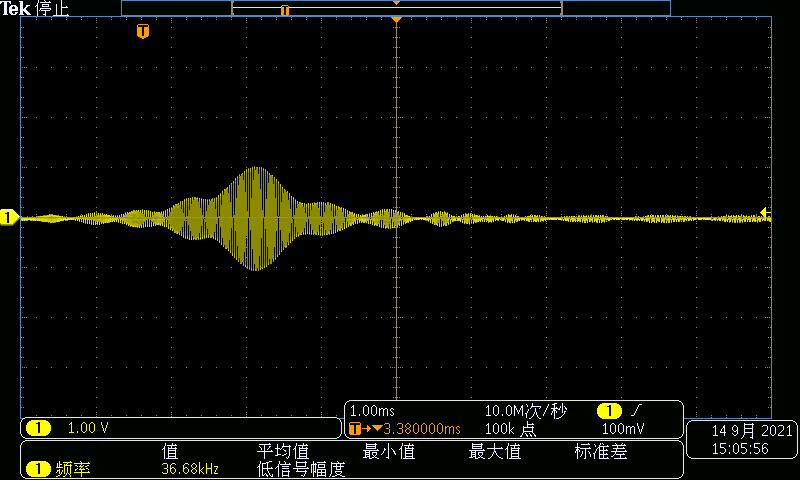

Supplement: Supplementary file 1 — Supplementary Information. [file 41598_2024_59328_MOESM1_ESM.zip › Decay experiment data/tek0034.png]

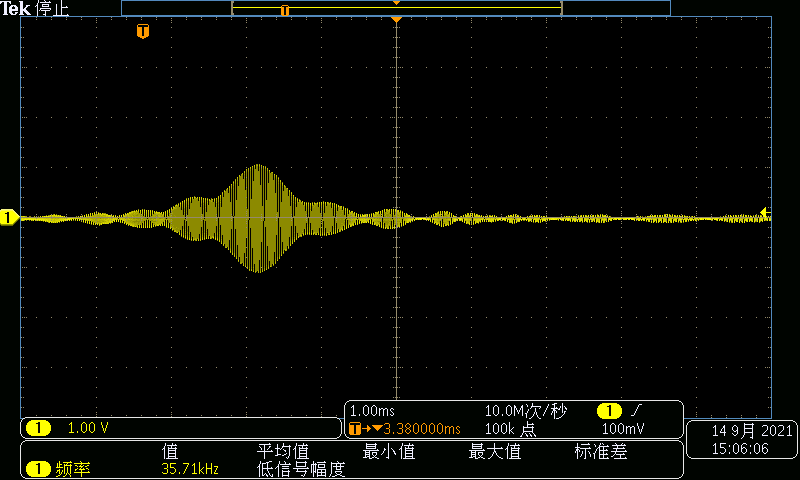

Supplement: Supplementary file 1 — Supplementary Information. [file 41598_2024_59328_MOESM1_ESM.zip › Decay experiment data/tek0035.png]

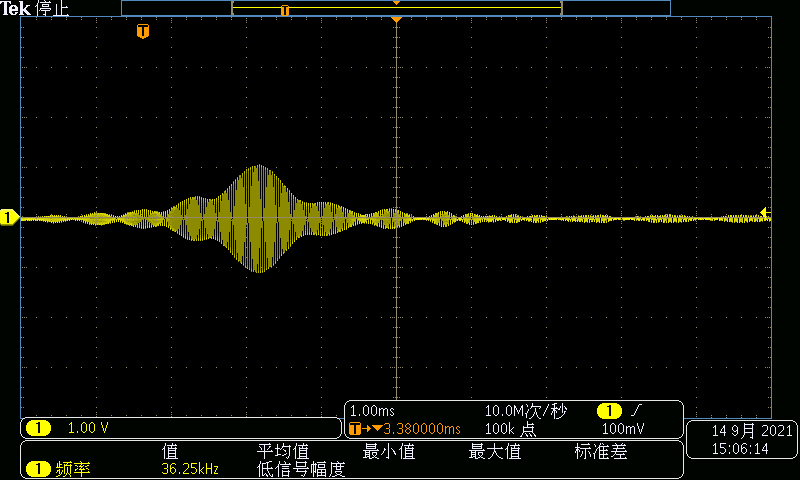

Supplement: Supplementary file 1 — Supplementary Information. [file 41598_2024_59328_MOESM1_ESM.zip › Decay experiment data/tek0036.png]

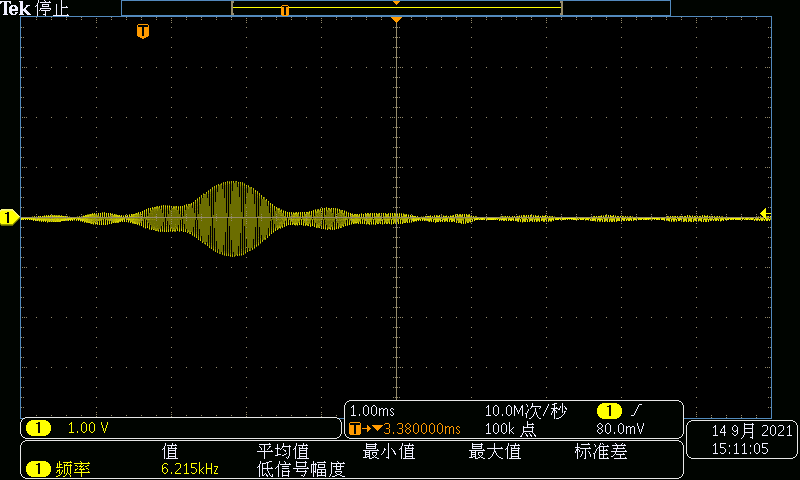

Supplement: Supplementary file 1 — Supplementary Information. [file 41598_2024_59328_MOESM1_ESM.zip › Decay experiment data/tek0037.png]

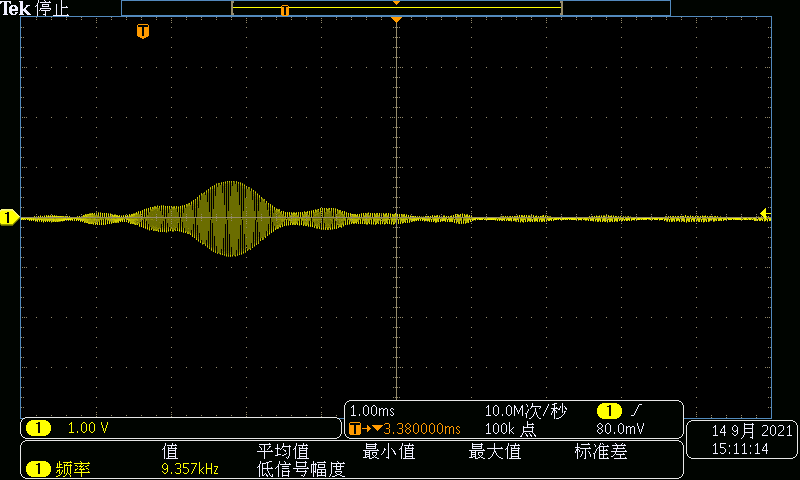

Supplement: Supplementary file 1 — Supplementary Information. [file 41598_2024_59328_MOESM1_ESM.zip › Decay experiment data/tek0038.png]

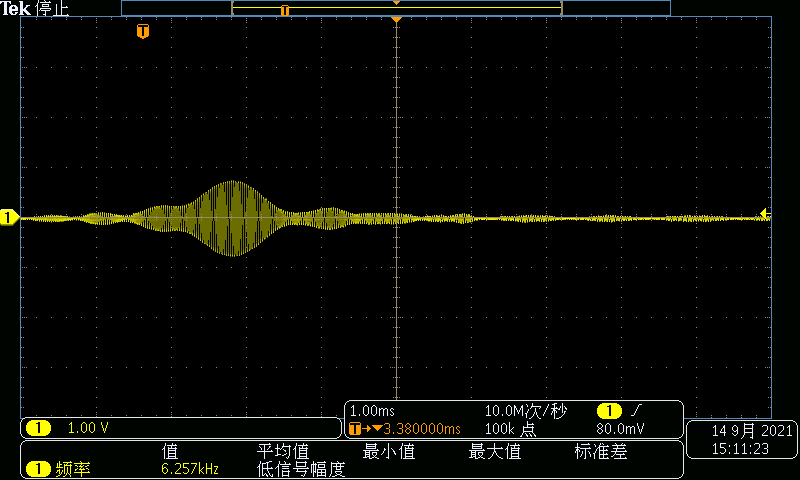

Supplement: Supplementary file 1 — Supplementary Information. [file 41598_2024_59328_MOESM1_ESM.zip › Decay experiment data/tek0039.png]

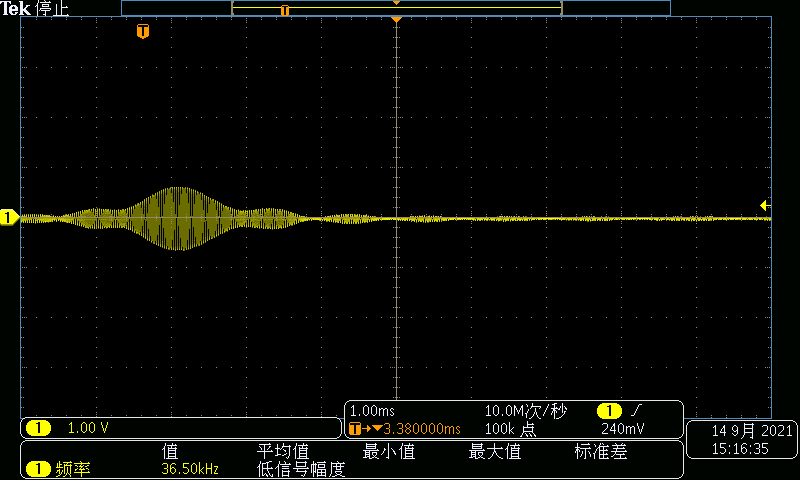

Supplement: Supplementary file 1 — Supplementary Information. [file 41598_2024_59328_MOESM1_ESM.zip › Decay experiment data/tek0040.png]

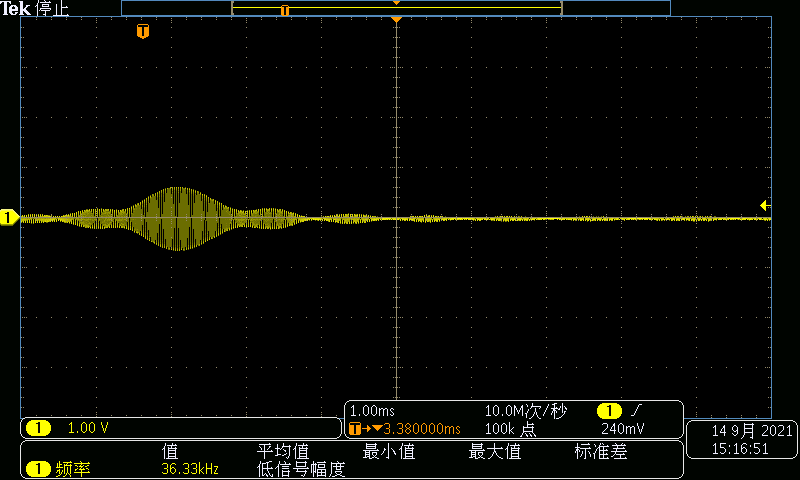

Supplement: Supplementary file 1 — Supplementary Information. [file 41598_2024_59328_MOESM1_ESM.zip › Decay experiment data/tek0041.png]

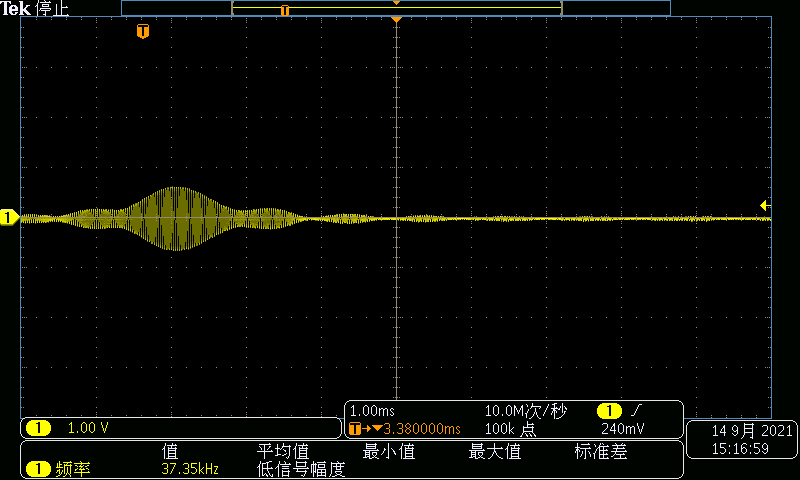

Supplement: Supplementary file 1 — Supplementary Information. [file 41598_2024_59328_MOESM1_ESM.zip › Decay experiment data/tek0042.png]

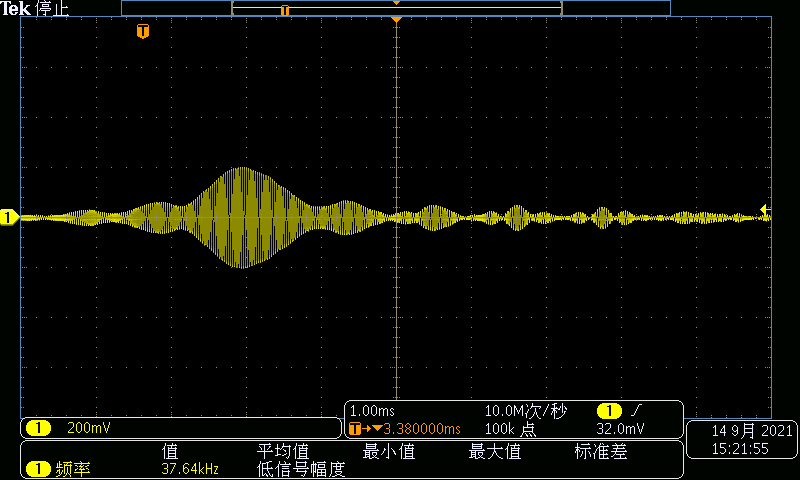

Supplement: Supplementary file 1 — Supplementary Information. [file 41598_2024_59328_MOESM1_ESM.zip › Decay experiment data/tek0043.png]

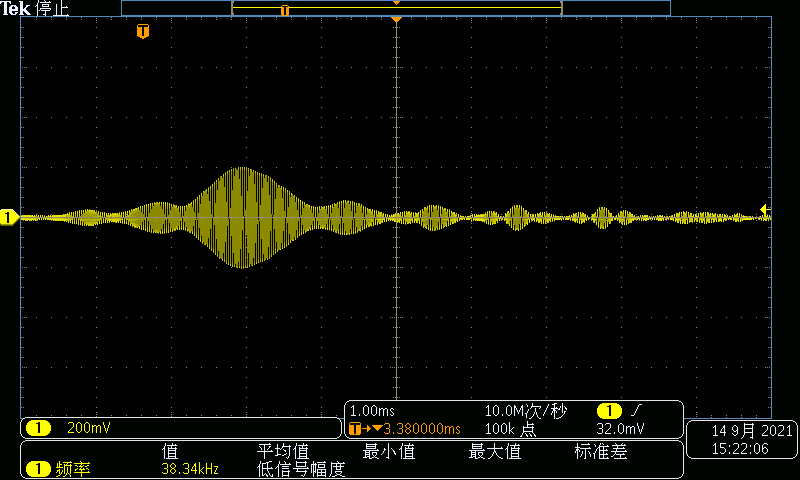

Supplement: Supplementary file 1 — Supplementary Information. [file 41598_2024_59328_MOESM1_ESM.zip › Decay experiment data/tek0044.png]

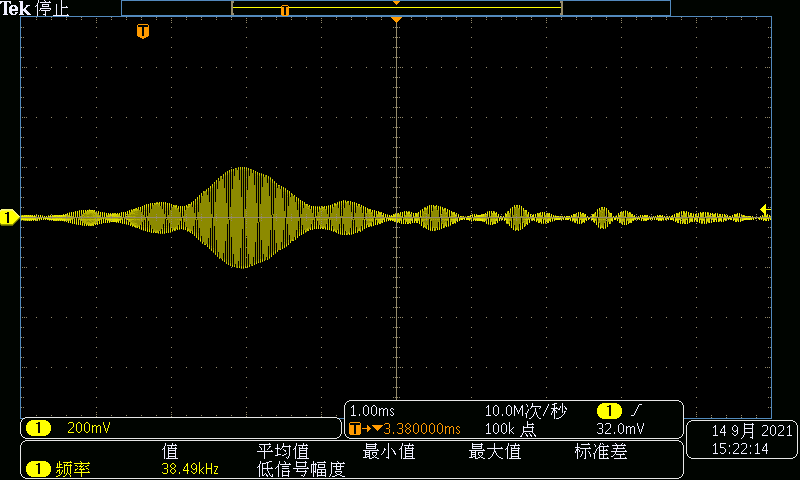

Supplement: Supplementary file 1 — Supplementary Information. [file 41598_2024_59328_MOESM1_ESM.zip › Decay experiment data/tek0045.png]

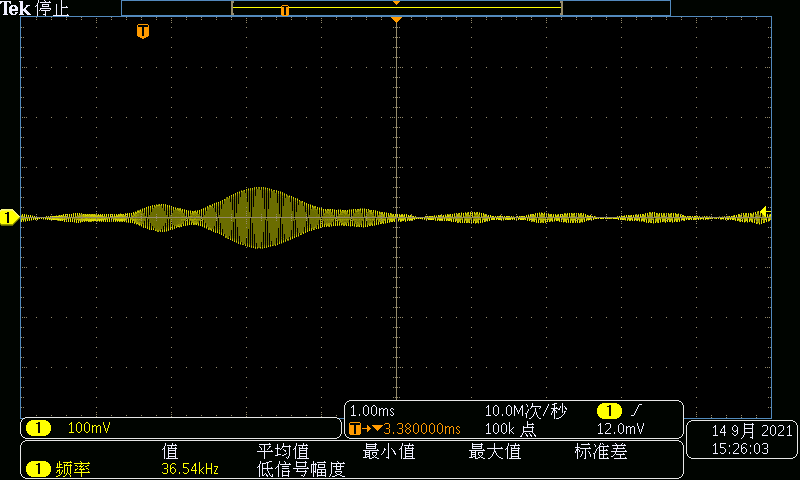

Supplement: Supplementary file 1 — Supplementary Information. [file 41598_2024_59328_MOESM1_ESM.zip › Decay experiment data/tek0046.png]

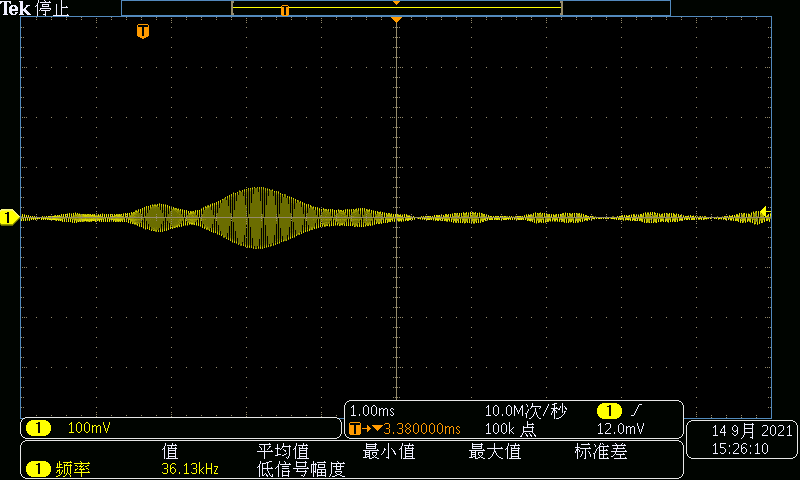

Supplement: Supplementary file 1 — Supplementary Information. [file 41598_2024_59328_MOESM1_ESM.zip › Decay experiment data/tek0047.png]

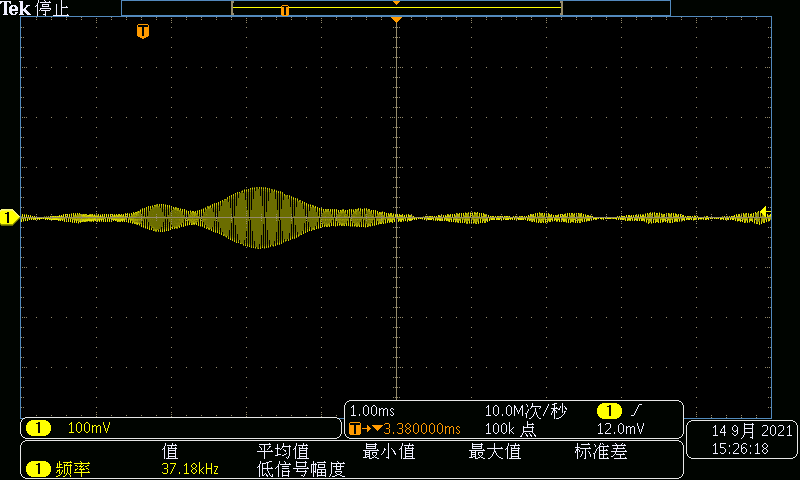

Supplement: Supplementary file 1 — Supplementary Information. [file 41598_2024_59328_MOESM1_ESM.zip › Decay experiment data/tek0048.png]

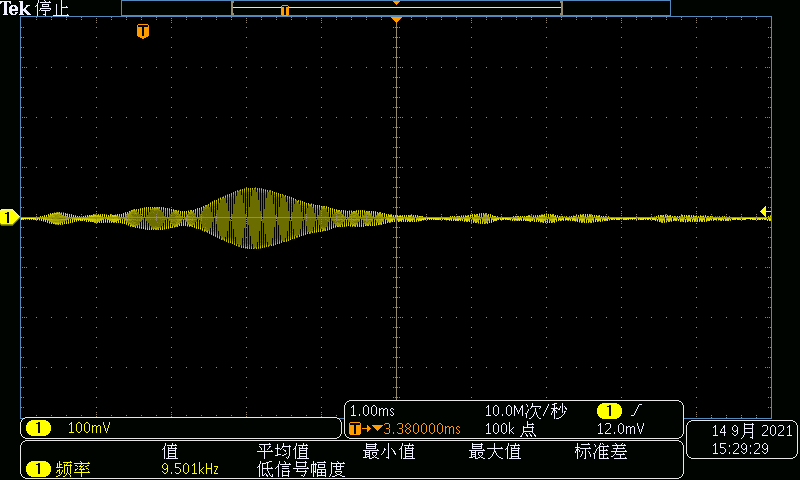

Supplement: Supplementary file 1 — Supplementary Information. [file 41598_2024_59328_MOESM1_ESM.zip › Decay experiment data/tek0049.png]

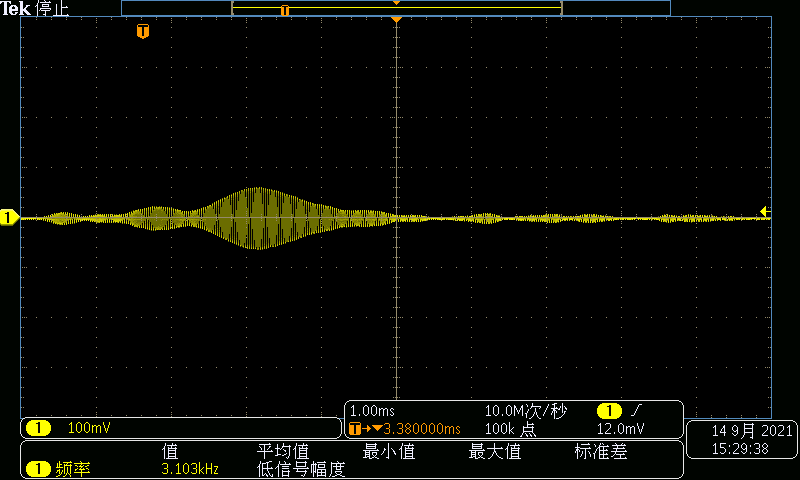

Supplement: Supplementary file 1 — Supplementary Information. [file 41598_2024_59328_MOESM1_ESM.zip › Decay experiment data/tek0050.png]

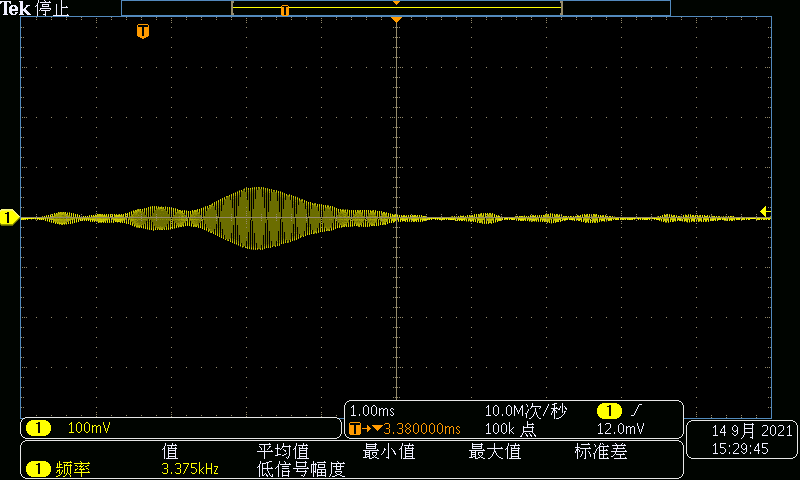

Supplement: Supplementary file 1 — Supplementary Information. [file 41598_2024_59328_MOESM1_ESM.zip › Decay experiment data/tek0051.png]

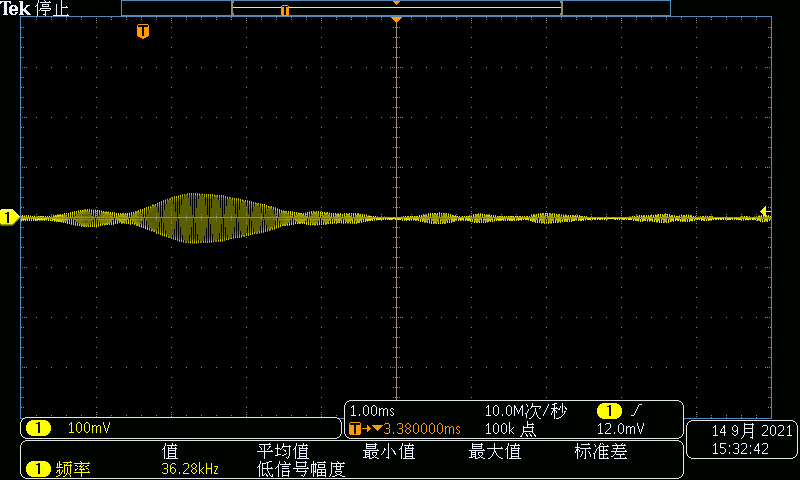

Supplement: Supplementary file 1 — Supplementary Information. [file 41598_2024_59328_MOESM1_ESM.zip › Decay experiment data/tek0052.png]

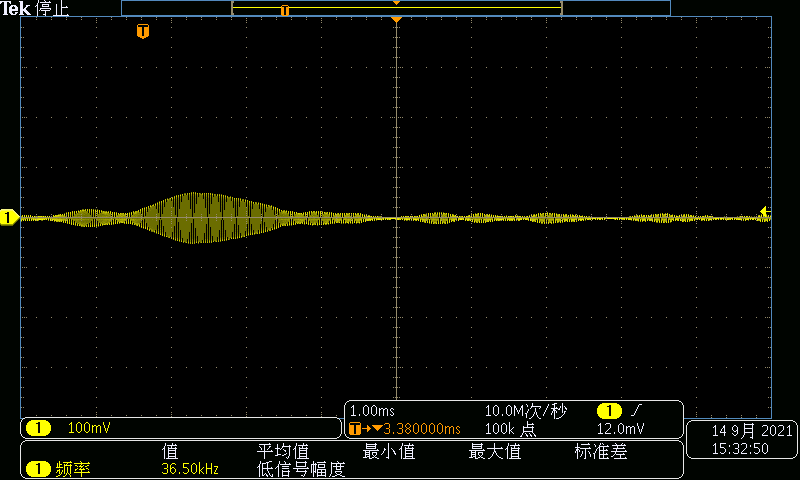

Supplement: Supplementary file 1 — Supplementary Information. [file 41598_2024_59328_MOESM1_ESM.zip › Decay experiment data/tek0053.png]

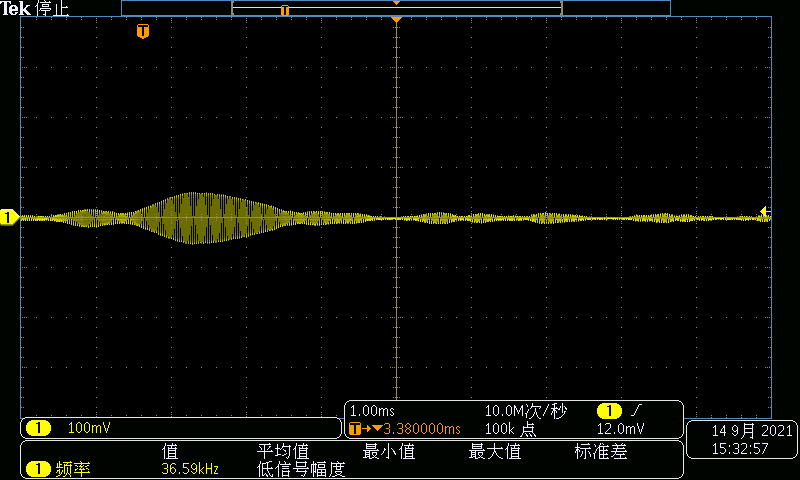

Supplement: Supplementary file 1 — Supplementary Information. [file 41598_2024_59328_MOESM1_ESM.zip › Decay experiment data/tek0054.png]

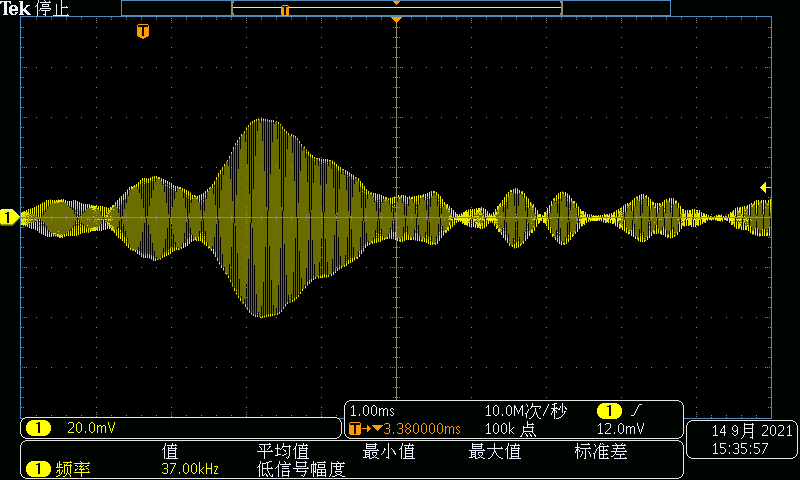

Supplement: Supplementary file 1 — Supplementary Information. [file 41598_2024_59328_MOESM1_ESM.zip › Decay experiment data/tek0055.png]

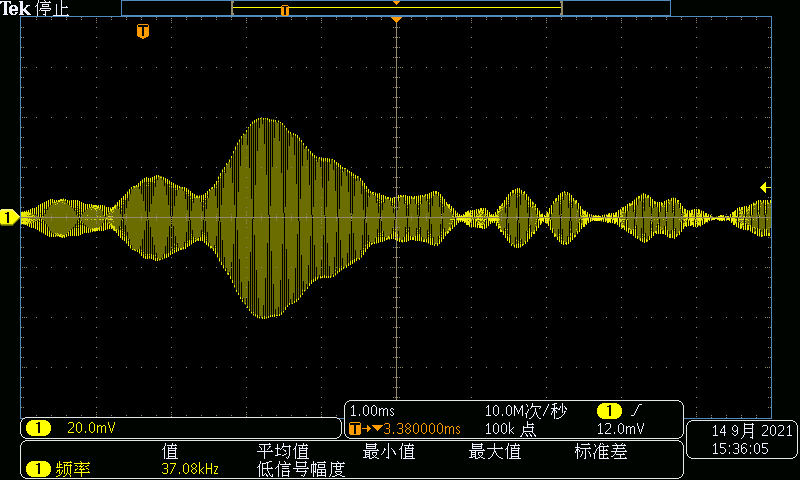

Supplement: Supplementary file 1 — Supplementary Information. [file 41598_2024_59328_MOESM1_ESM.zip › Decay experiment data/tek0056.png]

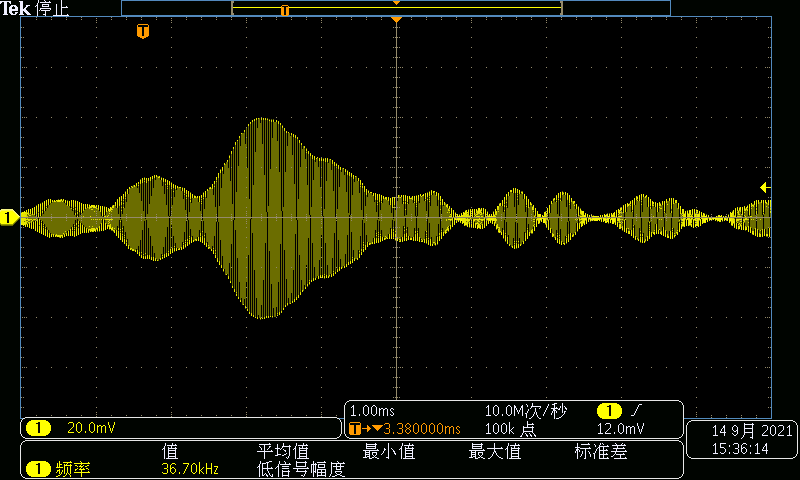

Supplement: Supplementary file 1 — Supplementary Information. [file 41598_2024_59328_MOESM1_ESM.zip › Decay experiment data/tek0057.png]

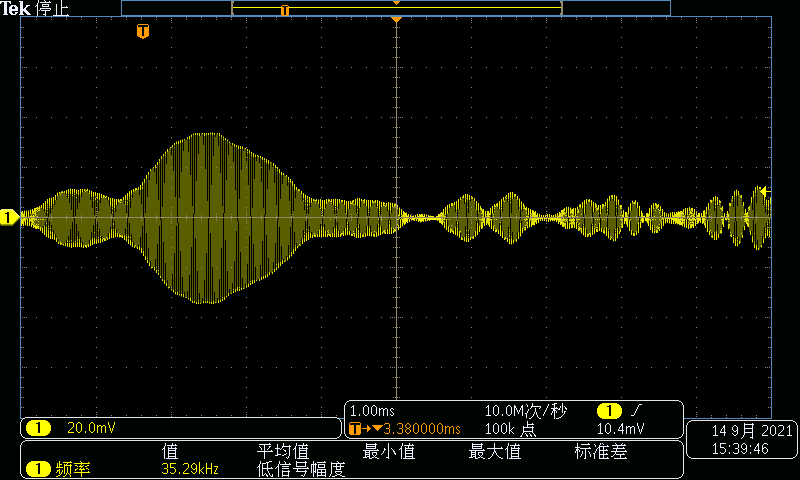

Supplement: Supplementary file 1 — Supplementary Information. [file 41598_2024_59328_MOESM1_ESM.zip › Decay experiment data/tek0058.png]

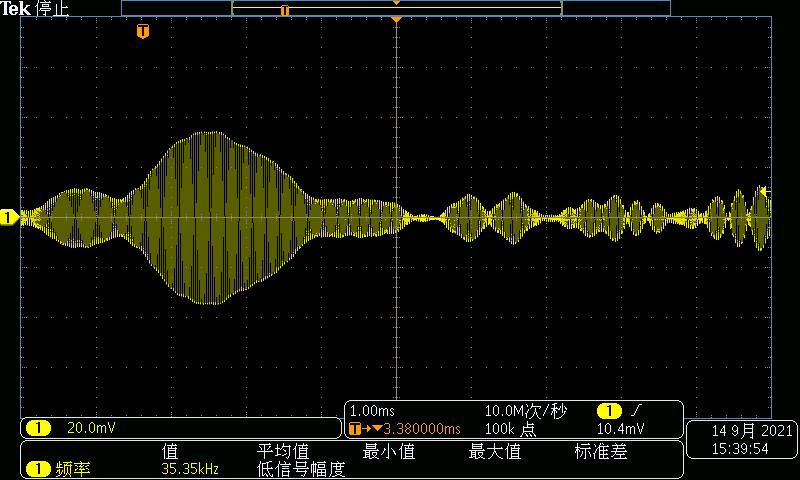

Supplement: Supplementary file 1 — Supplementary Information. [file 41598_2024_59328_MOESM1_ESM.zip › Decay experiment data/tek0059.png]

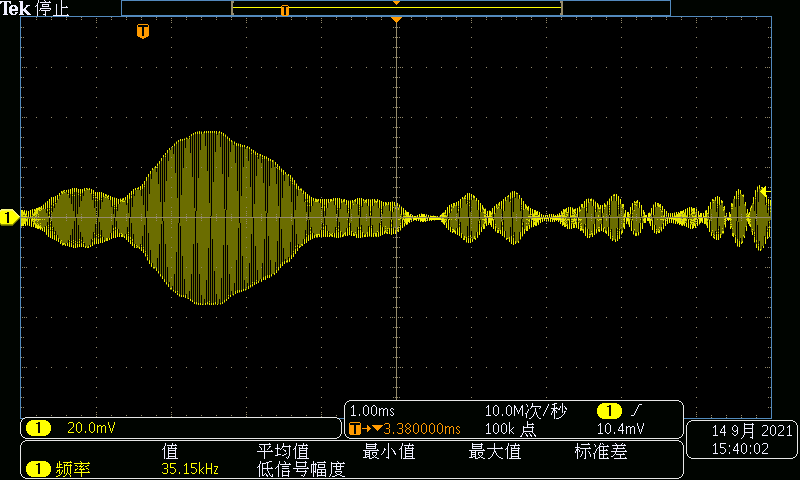

Supplement: Supplementary file 1 — Supplementary Information. [file 41598_2024_59328_MOESM1_ESM.zip › Decay experiment data/tek0060.png]

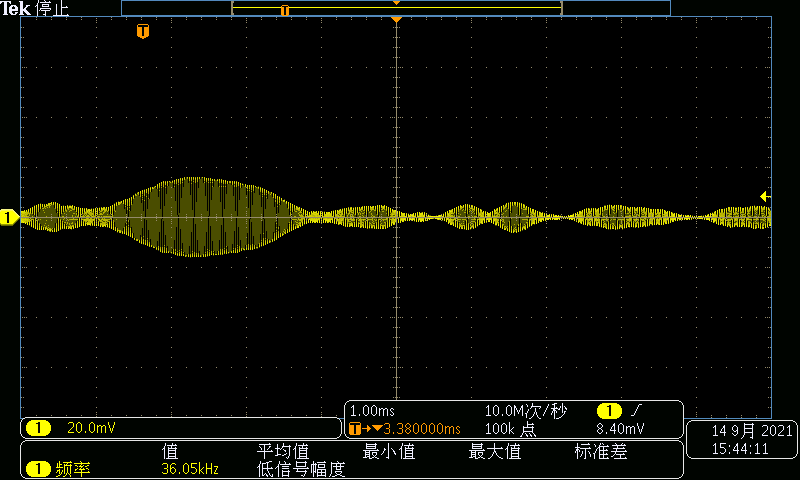

Supplement: Supplementary file 1 — Supplementary Information. [file 41598_2024_59328_MOESM1_ESM.zip › Decay experiment data/tek0061.png]
